# RNF115 Inhibits the Post‐ER Trafficking of TLRs and TLRs‐Mediated Immune Responses by Catalyzing K11‐Linked Ubiquitination of RAB1A and RAB13

**DOI:** 10.1002/advs.202105391

**Published:** 2022-03-28

**Authors:** Zhi‐Dong Zhang, Hong‐Xu Li, Hu Gan, Zhen Tang, Yu‐Yao Guo, Shu‐Qi Yao, Tianzi Liuyu, Bo Zhong, Dandan Lin

**Affiliations:** ^1^ Department of Gastrointestinal Surgery Medical Research Institute Zhongnan Hospital of Wuhan University Wuhan 430071 China; ^2^ Department of Pulmonary and Critical Care Medicine Zhongnan Hospital of Wuhan University Wuhan 430071 China; ^3^ Frontier Science Center for Immunology and Metabolism Wuhan University Wuhan 430071 China; ^4^ Cancer Center Renmin Hospital of Wuhan University Wuhan 430061 China; ^5^ Department of Virology College of Life Sciences Wuhan University Wuhan 430072 China; ^6^ Wuhan Research Center for Infectious Diseases and Cancer Chinese Academy of Medical Sciences Wuhan 430071 China

**Keywords:** intracellular trafficking, RAB proteins, RNF115, signaling transduction, Toll‐like receptors, ubiquitination

## Abstract

The subcellular localization and intracellular trafficking of Toll‐like receptors (TLRs) critically regulate TLRs‐mediated antimicrobial immunity and autoimmunity. Here, it is demonstrated that the E3 ubiquitin ligase RNF115 inhibits the post‐endoplasmic reticulum (ER) trafficking of TLRs and TLRs‐mediated immune responses by catalyzing ubiquitination of the small GTPases RAB1A and RAB13. It is shown that the 14‐3‐3 chaperones bind to AKT1‐phosphorylated RNF115 and facilitate RNF115 localizing on the ER and the Golgi apparatus. RNF115 interacts with RAB1A and RAB13 and catalyzes K11‐linked ubiquitination on the Lys49 and Lys61 residues of RAB1A and on the Lys46 and Lys58 residues of RAB13, respectively. Such a modification impairs the recruitment of guanosine diphosphate (GDP) dissociation inhibitor 1 (GDI1) to RAB1A and RAB13, a prerequisite for the reactivation of RAB proteins. Consistently, knockdown of RAB1A and RAB13 in *Rnf115* ^+/+^ and *Rnf115* ^−/−^ cells markedly inhibits the post‐ER and the post‐Golgi trafficking of TLRs, respectively. In addition, reconstitution of RAB1A^K49/61R^ or RAB13^K46/58R^ into *Rnf115* ^+/+^ cells but not *Rnf115* ^−/−^ cells promotes the trafficking of TLRs from the ER to the Golgi apparatus and from the Golgi apparatus to the cell surface, respectively. These findings uncover a common and step‐wise regulatory mechanism for the post‐ER trafficking of TLRs.

## Graphical abstract

Toll‐like receptors (TLRs) undergo ordered intracellular trafficking to subcellular compartments and plasma membrane where they recognize pathogen‐associated molecular patterns (PAMPs) for activation and signaling. Here, it is shown that RNF115 inhibits the endoplasmic reticulum (ER)‐Golgi and Golgi‐plasma membrane trafficking of TLRs by catalyzing ubiquitination of RAB1A and RAB13 respectively. These findings uncover a common and step‐wise regulatory mechanism for the post‐ER trafficking of TLRs.

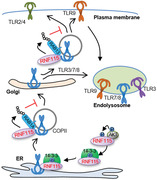

## 1 Introduction

Pattern‐recognition receptors (PRRs) detect microbial pathogen‐associated molecular patterns (PAMPs) and host‐derived danger‐associated molecular patterns (DAMPs) to initiate a series of signaling cascades that elicit antimicrobial immune responses or induce autoimmune disorders. Toll‐like receptors (TLRs) are transmembrane PRRs that recognize a wide range of PAMPs or DAMPs to induce immune responses.^[^ ^1^ ^]^ For example, TLR4 recognizes lipopolysaccharide (LPS) on the cell wall of Gram‐negative bacteria and TLR2 is activated by the cell wall component zymosan or lipoteichoic acid of Gram‐positive bacteria,^[^ ^2^, ^3^ ^]^ whereas TLR7 and TLR9 recognize single‐stranded RNA and unmethylated CpG DNA, respectively.^[^ ^4^, ^5^, ^6^, ^7^ ^]^ This specificity facilitates immune responses against a broad array of pathogens but introduces the potential for autoimmunity to commensal microbiota or self‐RNA and self‐DNA. Dysregulation of TLR4 or TLR2 signalingresults in hypersensitivity to dextran sulfate sodium (DSS)‐induced colitis and improper activation of TLR7 and TLR9 by self‐nucleic acids results in autoimmune disorders in mice.^[^ ^8^, ^9^, ^10^, ^11^, ^12^ ^]^ In addition, polymorphisms of TLRs are associated with various autoinflammatory diseases in humans, including inflammatory bowel diseases, rheumatoid arthritis, psoriasis, and systemic lupus erythematosus.^[^ ^13^, ^14^ ^]^ Therefore, understanding TLRs activation and signaling would help to decipher the mechanisms of diseases and provide potential therapeutic intervention strategies.

TLRs undergo multi‐step posttranslational regulations accompanied with ordered intracellular trafficking to subcellular compartments and plasma membrane where they recognize and bind to PMAPs or DAMPs for activation and signaling.^[^ ^15^, ^16^, ^17^ ^]^ Two ER‐localized chaperones gp96 and PRAT4A associate with TLRs on the ER for the proper folding and glycosylation of multiple TLRs before they exit from the ER.^[^ ^18^, ^19^, ^20^ ^]^ Simultaneously, a subset of TLRs (TLR3, 5, 7, 8, 9) binds to another ER chaperone UNC93B1 that stabilizes TLRs and promotes TLRs incorporation into coat protein complex II vesicles to bud off the transitional ER.^[^ ^21^, ^22^, ^23^, ^24^ ^]^ UNC93B1 further escorts these TLRs to the Golgi apparatus for additional glycosylation and to endolysosomal compartments for ectodomain cleavage which are prerequisites for the activation of TLRs.^[^ ^16^ ^]^ In contrast, the other subset of TLRs (TLR1, 2, 4, 6) leaves from the ER to the Golgi apparatus in an UNC93B1‐independent manner for further glycosylation and finally to reach cell surface.^[^ ^16^ ^]^ However, the mechanisms for the UNC93B1‐dependent and independent transport of TLRs from the ER to the Golgi apparatus, cell surface, or endolysosomes are unclear.

RNF115 has been shown highly expressed in invasive breast cancers and genome‐wide association studies have identified *RNF115* as a susceptible locus for breast cancer.^[^ ^25^, ^26^, ^27^, ^28^ ^]^ Recent studies have suggested that RNF115 promotes tumorigenesis and malignant cell migration in multiple murine tumor models.^[^ ^29^, ^30^, ^31^, ^32^ ^]^ Though it has been shown that RNF115 is involved in EGF receptor endosomal sorting and degradation,^[^ ^33^, ^34^ ^]^ knockout of RNF115 has no obvious effects on the breeding and development of mice.^[^ ^35^ ^]^ We have recently demonstrated that RNF115 constitutively interacts with and destabilizes MAVS under homeostatic conditions by catalyzing ubiquitination and degradation of MAVS. In contrast, RNF115 catalyzes ubiquitination of MITA for its oligomerization after HSV‐1 infection. Consequently, the *Rnf115* ^−/−^ mice exhibit increased and decreased resistance to EMCV or HSV‐1 infection compared to the wild‐type littermates, respectively.^[^ ^35^ ^]^ Whether and how RNF115 regulates TLRs‐mediated immune responses remain to be investigated.

In this study, we have demonstrated that knockout of RNF115 significantly promotes the trafficking of TLRs from the ER to subcellular compartments and cell surface, and thereby potentiates TLRs‐mediated signaling and TLRs‐induced immune responses. RNF115 is phosphorylated by AKT1 and subsequently associates with the 14‐3‐3 chaperones that facilitate RNF115 associating with TLRs and localizing on the ER and the Golgi apparatus. Furthermore, RNF115 catalyzes K11‐linked ubiquitination on the Lys49 and Lys61 residues of RAB1A and on the Lys46 and Lys58 residues of RAB13, which impairs the recruitment of GDP dissociation inhibitor 1 (GDI1) to RAB1A and RAB13, thereby inhibiting the reactivation of the two RABs and the post‐ER trafficking of TLRs. Specifically, RAB1A promotes the trafficking of TLRs from the ER to the Golgi apparatus, whereas RAB13 facilitates the trafficking of a subset of TLRs from the Golgi apparatus to the cell surface. These data indicate the AKT‐RNF115‐RAB axis as a common regulatory machinery for the post‐ER trafficking of TLRs.

## 2 Results

### 2.1 RNF115 Negatively Regulates TLR‐Induced Expression of Proinflammatory Cytokines

**Figure 1:**
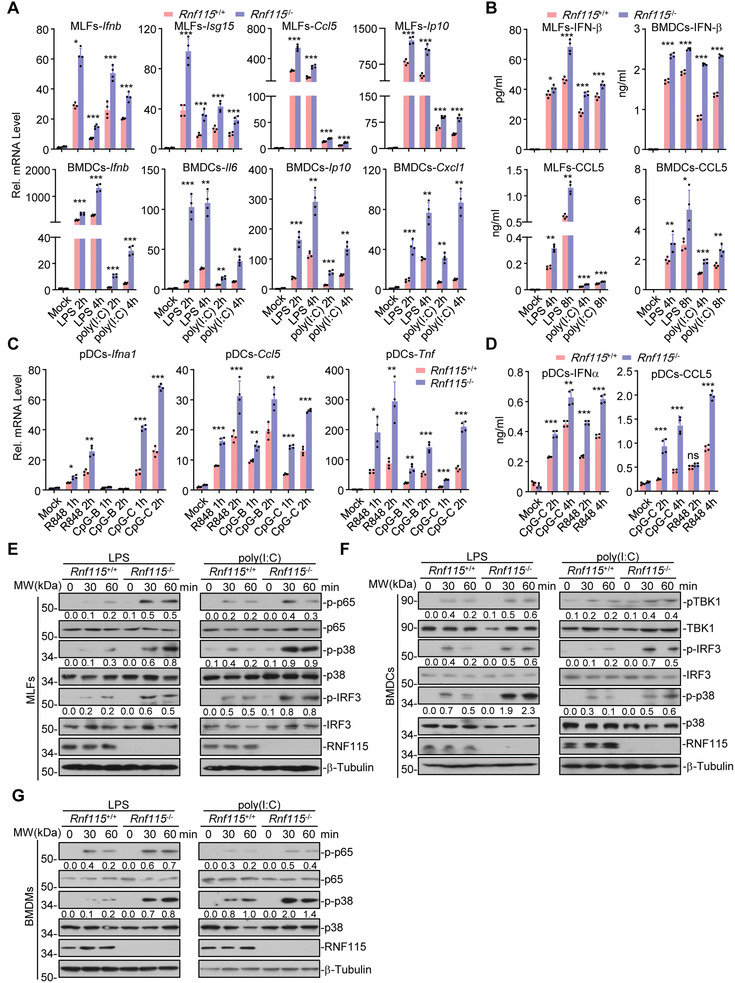
RNF115 negatively regulates TLR‐mediated signaling. A) qPCR analysis of *Ifnb, Isg15, Ip10, Il6, Cxcl1, or Ccl5* mRNA in *Rnf115* ^+/+^ and *Rnf115* ^−/−^ murine lung fibroblasts (MLFs) or bone marrow‐derived dendritic cells (BMDCs) stimulated with LPS (1 µg mL^−1^) or poly(I:C) (50 µg mL^−1^) for 0, 2, or 4 h. B) ELISA analysis of IFN‐*β* and CCL5 in the supernatants of *Rnf115* ^+/+^ and *Rnf115* ^−/−^ MLFs or BMDCs stimulated with LPS (1 µg mL^−1^) or poly(I:C) (50 µg mL^−1^) for 0–8 h. C) qPCR analysis of *Ifna1, Tnf, or Ccl5* mRNA in *Rnf115* ^+/+^ and *Rnf115* ^−/−^ pDCs left stimulated with R848 (1 µg mL^−1^), CpG‐B (5 × 10^−6^ m), or CpG‐C (5 × 10^−6^ m) for 0, 1, or 2 h. D) ELISA analysis of IFN‐*α* and CCL5 in the supernatants of *Rnf115* ^+/+^ and *Rnf115* ^−/−^ Flt3L‐generated plasmacytoid dendritic cells (pDCs) left stimulated with R848 (1 µg mL^−1^) or CpG‐C (5 × 10^−6^ m) for 0, 2, or 4 h. E,F) Immunoblot analysis of total and phosphorylated E) (p‐) p65, IRF3, and p38, total RNF115 and *β*‐Tubulin in *Rnf115* ^+/+^ and *Rnf115* ^−/−^ MLFs or F) BMDCs stimulated with LPS (1 µg mL^−1^) or poly(I:C) (50 µg mL^−1^) for 0–60 min. G) Immunoblot analysis of total and phosphorylated (p‐) p65 and p38, total RNF115 and *β*‐Tubulin in *Rnf115* ^+/+^ and *Rnf115* ^−/−^ BMDMs stimulated with LPS (1 µg mL^−1^) or poly(I:C) (50 µg mL^−1^) for 0–60 min. *p < 0.05, **p < 0.01, and ***p < 0.001 (two‐tailed Student's *t*‐test). Data are representative of three independent experiments (graphs show mean ± SD in (A–D)).

TLRs are type 1 transmembrane pattern recognition receptors that induce the expression of proinflammatory cytokines and inflammatory responses after binding to their ligands.^[^ ^1^ ^]^ To examine whether the E3 ubiquitin ligase RNF115 played a role in TLR‐induced expression of proinflammatory cytokines, we have stimulated the primary murine *Rnf115* ^+/+^ and *Rnf115* ^−/−^ cells with various TLR ligands followed by quantitative RT‐PCR (qPCR) assays. The results suggested that knockout of RNF115 potentiated the expression or type I interferons (IFNs) and proinflammatory cytokines such as *Cxcl1*, *Ip10*, and *Ccl5* in mouse fibroblasts (MLFs) and bone marrow‐derived dendritic cells (BMDCs) after LPS, poly(I:C), Pam_3_CSK_4_ or PGN stimulation (**Figure** **1**A,B; Figure S1A, Supporting Information). In addition, R848‐ or CpG‐induced expression and production of IFN‐*β* and CCL5 was significantly higher in *Rnf115* ^−/−^ Flt3L‐generated plasmacytoid dendritic cells (pDCs) than in *Rnf115* ^+/+^ pDCs (Figure 1C,D). In contrast, knockout of RNF115 did not affect IFN‐*α*‐ or IFN‐*β*‐induced expression of *Isg15*, *Oas2*, or *Mx1* in MLFs (Figure S1B, Supporting Information). In addition, TNF‐ or IL‐1*β*‐induced expression of *Ip10*, *Cxcl1*, and *Il6* and production of CXCL1, IL‐6, and CCL5 were comparable between *Rnf115* ^+/+^ and *Rnf115* ^−/−^ MLFs (Figure S1B,C, Supporting Information). These data suggest that RNF115 selectively regulates TLR ligands‐induced expression of proinflammatory cytokines.

The phosphorylation of MAPKs, NF‐*κ*B, and/or IRF3 is a hallmark of TLR‐induced signaling cascades. Consistent with the results from the gene induction analyses, we found that LPS‐ or poly(I:C)‐induced phosphorylation of p65, p38, and IRF3 was substantially enhanced in *Rnf115* ^−/−^ MLFs, BMDCs, or BMDMs compared to the *Rnf115* ^+/+^ counterparts (Figure 1E–G), indicating an inhibitory role of RNF115 in TLR‐induced signaling cascades.

The E3 ubiquitin ligase activity of RNF115 is essential for its regulation of antiviral immunity.^[^ ^35^ ^]^ Similarly, we found that reconstitution of wild‐type RNF115 but not the inactive mutant RNF115(C228A/C231A) (RNF115^2CA^) into *Rnf115* ^−/−^ MLFs inhibited poly(I:C)‐ or LPS‐induced expression of *Ifnb*, *Ccl5*, and *Tnf* or phosphorylation of IRF3, TBK1, and p38 (Figure S1D–F, Supporting Information). These data collectively suggest that RNF115 negatively regulates innate immune signaling mediated by a broad subset of TLRs in a manner dependent on its ubiquitin ligase activity.

### 2.2 Knockout of RNF115 Potentiates TLR‐Induced Immune Responses in Mice

We next examined whether RNF115 regulated TLR‐induced immune responses in vivo. The *Rnf115* ^+/+^ and *Rnf115* ^−/−^ mice were intraperitoneally (i.p.) injected with LPS or intravenously (i.v.) injected with R848 or CpG‐B followed by various analyses (Figure S2A, Supporting Information). The results suggested that the *Rnf115* ^−/−^ mice were more susceptible to LPS‐induced septic shock than the *Rnf115* ^+/+^ mice (Figure S2B, Supporting Information). Consistent with the observations, the *Rnf115* ^−/−^ mice produced higher levels of TNF and CCL5 in the sera than the *Rnf115* ^+/+^ mice after LPS injection (Figure S2C, Supporting Information). The inflammation in the lungs from *Rnf115* ^−/−^ mice was more severe than those from *Rnf115* ^+/+^ mice after LPS injection as indicated by HE staining (Figure S2D, Supporting Information). In addition, the mRNA levels of proinflammatory cytokines (*Il6*, *Cxcl1*, *Tnf*) in the blood cells and the protein levels of type I IFNs and proinflammatory cytokines such as CXCL1 and CCL5 in the sera were significantly enhanced in *Rnf115* ^−/−^ mice compared to those in *Rnf115* ^+/+^ mice after injection of R848 or CpG‐B (Figure S2E–H, Supporting Information). These data suggest that RNF115 negatively regulates TLR‐induced production of proinflammatory cytokines in vivo.

**Figure 2:**
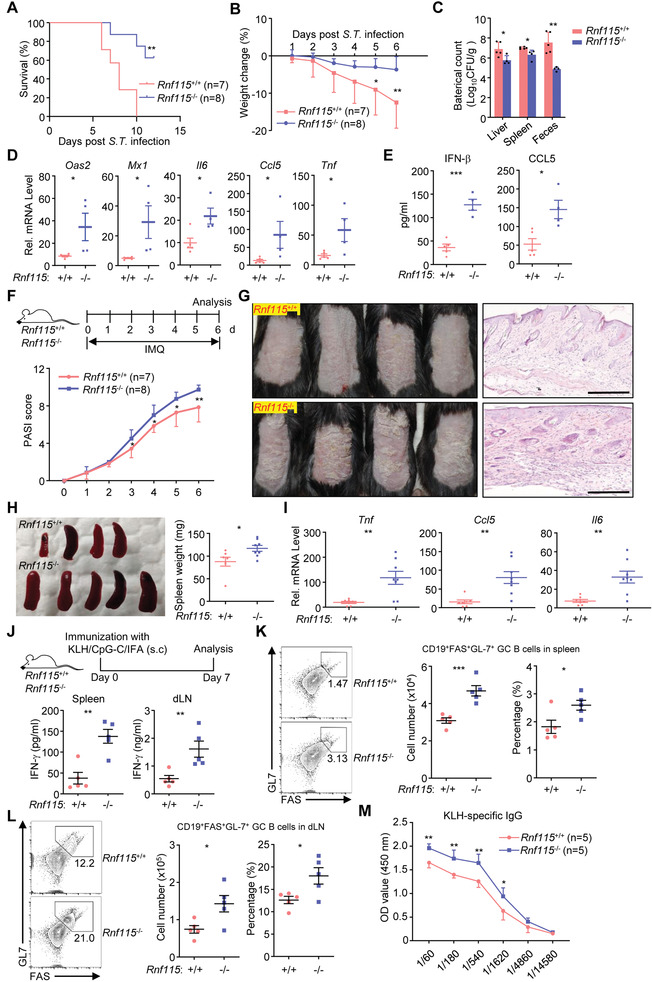
Knockout of RNF115 potentiates TLR‐induced immune responses. A) Survival (Kaplan‐Meier curve) of *Rnf115* ^+/+^ (*n* = 7) and *Rnf115* ^−/−^ mice (*n* = 8) injected with *Salmonellaenterica* Typhimurium (SL1344) (ST) (1 × 10^7^ CFU) by gavage and monitored for 14 d. B) Body weight change of *Rnf115* ^+/+^ (*n* = 7) and *Rnf115* ^−/−^ mice (*n* = 8) that were injected with ST (1 × 10^7^ CFU) by gavage. C) Bacterial counts in feces, liver, or spleen homogenates, D) qPCR analysis of the colon tissues, or E) ELISA analysis of indicated inflammatory cytokines in the sera of *Rnf115* ^+/+^ (*n* = 5) and *Rnf115* ^−/−^ mice (*n* = 4) that were injected with ST (1 × 10^7^ CFU) by gavage and monitored for 5 d. F) A scheme for Imiquimod (IMQ) treatment and PASI scores, G) hematoxylin–eosin (HE) staining of the back skin, H) spleen image and weight, or I) qPCR analysis of the back skins in *Rnf115* ^+/+^ (*n* = 7) and *Rnf115* ^−/−^ (*n* = 8) mice treated with a daily topical dose of IMQ cream for 6 consecutive days. J) *Rnf115* ^+/+^ and *Rnf115* ^−/−^ mice were subcutaneously (s.c.) immunized with KLH plus CpG‐C emulsified in incomplete Freud's adjuvant (IFA). Leukocytes of the spleen (3 × 10^6^) and draining lymph nodes (dLNs) (1 × 10^6^) were collected on day 7 and restimulated with KLH (5 mg mL^−1^) for 24 h. The secretion of IFN‐*γ* in the supernatants was measured by ELISA. (K, L) Flow cytometry to analyze the K) germinal center (GC) B cells in the spleen or L) dLNs from the *Rnf115* ^+/+^ and *Rnf115* ^−/−^ mice that were treated as in (J). M) The concentration of KLH‐specific IgG in the sera from the *Rnf115* ^+/+^ and *Rnf115* ^−/−^ mice that were treated as in (J). *p < 0.05, **p < 0.01, and ***p < 0.001 (two‐tailed Student's *t*‐test). Scale bars represent 200 µm. Data are combined of two independent experiments (A,B,F,H,I) or representative of two independent experiments (C–E, G, J–M) (graphs show mean ± SD in (A–F, H–M)).

Dysregulation of TLR signaling leads to various infectious diseases or autoimmune disorders.^[^ ^16^ ^]^ Consistent with the above observations that knockout of RNF115 promoted LPS‐induced production of proinflammatory cytokines in vivo, knockout of RNF115 resulted in resistance to the lethal infection of *Salmonella typhimurium* and *S. typhimurium*‐induced weight loss (**Figure** **2**A,B). The bacteria counts in the feces, liver, and spleens of *Rnf115* ^−/−^ mice were significantly decreased compared to those of *Rnf115* ^+/+^ mice (Figure 2C), whereas the expression of proinflammatory cytokines (*Il6*, *Tnf*, and *Ccl5*) in the colon and the production of IFN‐*β* and CCL5 in the sera of *Rnf115* ^−/−^ mice were increased compared to the counterparts of *Rnf115* ^+/+^ mice (Figure 2D,E). Imiquimod (IMQ) is a TLR7/8 agonist that exacerbates psoriasis in patients and induces psoriasis‐like lesions when topically applied to the shaved skin of mice.^[^ ^36^, ^37^, ^38^, ^39^ ^]^ Intriguingly, the severity of skin inflammation was aggravated in *Rnf115* ^−/−^ mice compared to the *Rnf115* ^+/+^ mice after IMQ treatment (Figure 2F,G). Consistently, the spleens were more enlarged and the expression levels of proinflammatory cytokines (*Tnf*, *Il6*, and *Ccl5*) were higher in the skin of IMQ‐treated *Rnf115* ^−/−^ mice than in the counterparts from *Rnf115* ^+/+^ mice (Figure 2H,I). Taken together, these data suggest that RNF115 negatively regulates TLR‐mediated antimicrobial immune responses and autoimmunity in vivo.

### 2.3 RNF115 Attenuates CpG‐Adjuvanted Vaccination

The synthetic CpG oligodeoxynucleotides (CpG‐ODN) and the related derivatives are immunostimulatory adjuvants that activate TLR9.^[^ ^40^ ^]^ Since RNF115 negatively regulated TLR9‐mediated signaling in cells and in mice, we further examined the role of RNF115 in vaccination with CpG‐ODN as an adjuvant. The *Rnf115* ^+/+^ and *Rnf115* ^−/−^ mice were subcutaneously (s.c.) injected with Keyhole limpet hemocyanin (KLH) emulsified in incomplete Freund's adjuvant (IFA) containing CpG‐C for seven days followed by various assays (Figure 2J). Importantly, the lymphocytes in the spleens and the draining lymph nodes of *Rnf115* ^−/−^ mice produced much higher IFN‐*γ* than those of *Rnf115* ^+/+^ mice after restimulation with KLH in vitro (Figure 2J). In addition, the percentages and numbers of GL7^+^FAS^+^ germinal center (GC) B cells were significantly increased in the spleens and the draining lymph nodes from *Rnf115* ^−/−^ mice compared to those from *Rnf115* ^+/+^ mice after immunization (Figure 2K,L). Consistently, the KLH‐specific IgG in the sera of *Rnf115* ^−/−^ mice was significantly higher than that of *Rnf115* ^+/+^ mice (Figure 2M). These results indicate that RNF115 limits vaccination responses mediated by TLR9 ligands.

### 2.4 Knockout of RNF115 Promotes the Glycosylation of TLRs

Because RNF115 regulated signaling triggered by a broad subset of TLRs and the E3 ubiquitin ligase activity was required for the function, we examined whether RNF115 interacted with and ubiquitinated TLRs. As shown in Figure S3A,B in the Supporting Information, RNF115 interacted with multiple TLRs but did not increase the ubiquitination of TLR4 or TLR9. In addition, the mRNA levels of *Tlr3*, *Tlr4*, *Tlr7*, or *Tlr9* were comparable between *Rnf115* ^+/+^ and *Rnf115* ^−/−^ BMDMs or *Rnf115* ^+/+^ and *Rnf115* ^−/−^ BMDCs (Figure S3C, Supporting Information). The ER chaperon gp96 and UNC93B1 is essential for the proper folding and transportation of TLRs respectively.^[^ ^20^, ^23^ ^]^ Though RNF115 interacted with gp96 and UNC93B1, it did not increase the ubiquitination of gp96 and UNC93B1 (Figure S3A,B, Supporting Information), indicating that TLRs and gp96 are unlikely the direct substrates of RNF115.

**Figure 3:**
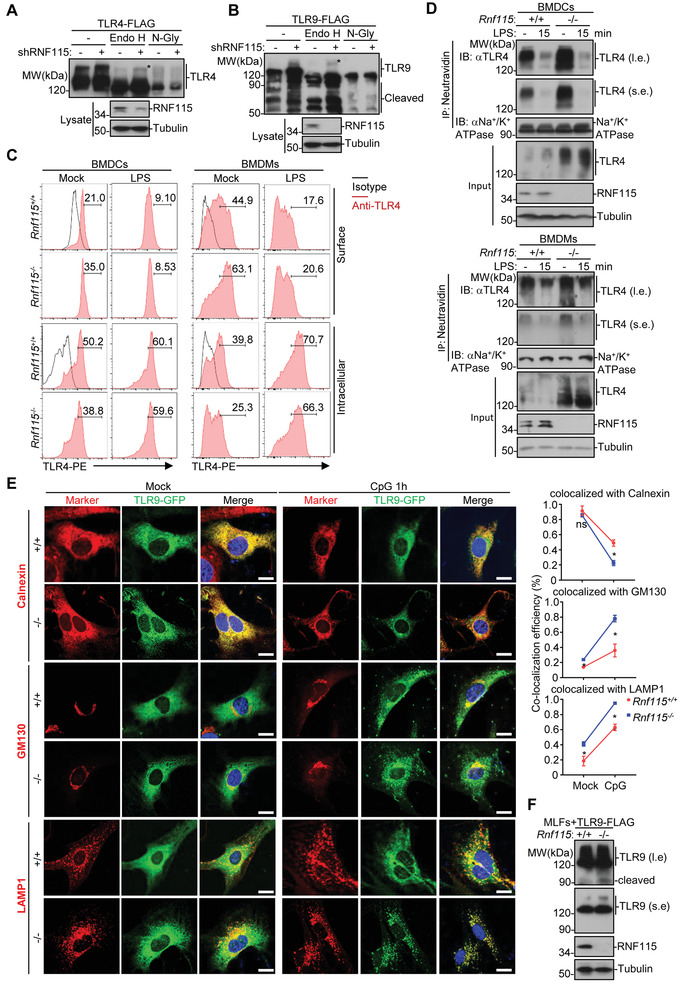
Knockout of RNF115 promotes the glycosylation and the post‐ER trafficking of TLRs. A,B) HEK293 cells that were transfected with the plasmids encoding A) TLR4‐FLAG or B) TLR9‐FLAG together with a control shRNA or shRNF115 for 36 h were lyzed and subjected to immunoprecipitation with anti‐FLAG (M2) agarose. The immunoprecipitates were washed and treated with buffer (‐), Endo H, or N‐glycanase (N‐Gly) for 1 h followed by immunoblot analysis with anti‐FLAG. The expression levels of RNF115 and Tubulin were analyzed by immunoblot analysis with the indicated antibodies. Endo H‐resistant forms of TLR4 and TLR9 were indicated with a star. C) Flow cytometry analysis of the surface and the intracellular TLR4 of *Rnf115* ^+/+^ and *Rnf115* ^−/−^ BMDCs or BMDMs that were left untreated or stimulated with LPS (1 µg mL^−1^) for 15 min followed by staining with anti‐TLR4‐PE (red line) or an isotype control (black line). D) *Rnf115* ^+/+^ and *Rnf115* ^−/−^ BMDCs or BMDMs were left unstimulated or stimulated with LPS (1 µg mL^−1^) for 15 min followed by incubation with biotin for 30 min. The nonbinding biotin was quenched by glycine for 20 min and the cell lysate was prepared followed by immunoprecipitation (with Neutraavidin agarose) and immunoblot analysis (with anti‐TLR4, anti‐Na^+^/K^+^ ATPase, anti‐RNF115, or antitubulin). l.e., long‐time exposure. s.e., short‐time exposure. E) Immunofluorescent staining of Calnexin (red), GM130 (red), or LAMP1 (red) and confocal microscopy analysis in *Rnf115* ^+/+^ and *Rnf115* ^−/−^ MLFs reconstituted with TLR9‐GFP left unstimulated or stimulated with CpG‐B (5 × 10^−6^ m) for 1 h. F) Immunoblot analysis (with anti‐FLAG, anti‐RNF115, or antitubulin) in *Rnf115* ^+/+^ and *Rnf115* ^−/−^ MLFs reconstituted with TLR9‐FLAG. *p < 0.05, **p < 0.01, ns, not significant (two‐tailed Student's *t*‐test). Scale bars represent 5 µm. Data are representative of three independent experiments (graphs show mean ± SD in (E)).

Interestingly, we found that overexpression decreased the molecular weight (MW) of TLR4 and TLR9 (Figure S3D, Supporting Information), whereas knockdown of RNF115 increased the MW of TLR3, TLR4, and TLR9 and potentiated the cleavage of TLR3 and TLR9 (Figure S3E, Supporting Information). Such modifications of the high MW of TLR4 or TLR9 were glycosylation at the Golgi apparatus, as they were insensitive to endoglycosidase H (Endo H) (which cleaves the N‐linked oligosaccharides on target proteins generated in the ER but not the more mature oligosaccharides generated in the Golgi apparatus) treatment but sensitive to N‐glycosidase F (N‐Gly) (which cleaves both oligosaccharides on proteins generated in the ER and the Golgi apparatus) treatment (**Figure** **3**A,B). These data collectively suggest that RNF115 negatively regulates the glycosylation and the cleavage of TLRs.

### 2.5 Knockout of RNF115 Facilitates the Post‐ER Trafficking of TLRs

The glycosylated TLR4 and TLR2 at the Golgi apparatus are transported to cell surface where they bind to their ligands and initiate signaling.^[^ ^16^ ^]^ Consistent with this notion, we observed that the cell surface TLR4 was increased and the intracellular TLR4 was decreased in *Rnf115* ^−/−^ BMDCs or BMDMs compared to *Rnf115* ^+/+^ BMDCs or BMDMs, respectively (Figure 3C,D). In addition, reconstitution of wild‐type RNF115 but not RNF115^2CA^ into *Rnf115* ^−/−^ MLFs decreased the surface TLR4 expression (Figure S3F, Supporting Information), indicating that RNF115 restricts the transportation of TLR4 to the cell surface in a manner dependent on its enzymatic activity. Similarly, the cell surface TLR2 was increased and the intracellular TLR2 was decreased in *Rnf115* ^−/−^ BMDCs or BMDMs compared to *Rnf115* ^+/+^ BMDCs or BMDMs, respectively (Figure S3G,H, Supporting Information). In contrast, however, the MHC‐I and the integrin molecules CD11b or CD11c on the cell surface were comparable between *Rnf115* ^+/+^ and *Rnf115* ^−/−^ BMDCs (Figure S3I, Supporting Information), indicating that RNF115 selectively inhibits the localization of TLR4 and TLR2 on the cell surface.

Treatment with LPS downregulated the cell surface TLR4 and increased the intracellular TLR4 in *Rnf115* ^+/+^ and *Rnf115* ^−/−^ BMDCs (Figure 3C,D). In addition, the levels of the cell surface TLR4 and the intracellular TLR4 were comparable between *Rnf115* ^+/+^ and *Rnf115* ^−/−^ BMDCs after LPS treatment (Figure 3C,D). The total levels of TLR4 in *Rnf115* ^+/+^ and *Rnf115* ^−/−^ BMDCs remained unchanged before and after LPS treatment (Figure 3D). Similarly, treatment with Pam_3_CSK_4_ downregulated the surface TLR2 and increased the intracellular TLR2 in *Rnf115* ^+/+^ BMDCs to a comparable level of those in *Rnf115* ^+/+^ BMDCs (Figure S3G, Supporting Information). These data support the notion that knockout of RNF115 does not inhibit ligand‐induced downregulation of the surface TLR4 and TLR2.

The TLR9 traffics through the Golgi apparatus en route to endolysosomes where TLR9 undergoes ectodomain cleavage.^[^ ^22^ ^]^ We found that there was increased co‐localization efficiency between TLR9‐GFP and GM130 or LAMP1 in *Rnf115* ^−/−^ MLFs compared to that in *Rnf115* ^+/+^ MLFs reconstituted with TLR9‐GFP in the presence or absence of CpG stimulation (Figure 3E), indicating that RNF115 restricts the transportation of TLR9 from the ER to the Golgi apparatus and the endolysosomal compartments. Consistent with these observations, the glycosylation and the cleavage of TLR9 were enhanced in *Rnf115* ^−/−^ MLFs compared to *Rnf115* ^+/+^ MLFs reconstituted with TLR9‐FLAG (Figure 3F). These data together suggest that RNF115 inhibits the post‐ER trafficking, glycosylation and cleavage of TLR9.

### 2.6 The 14‐3‐3 Chaperones Bind to AKT1‐Phosphorylated RNF115

**Figure 4:**
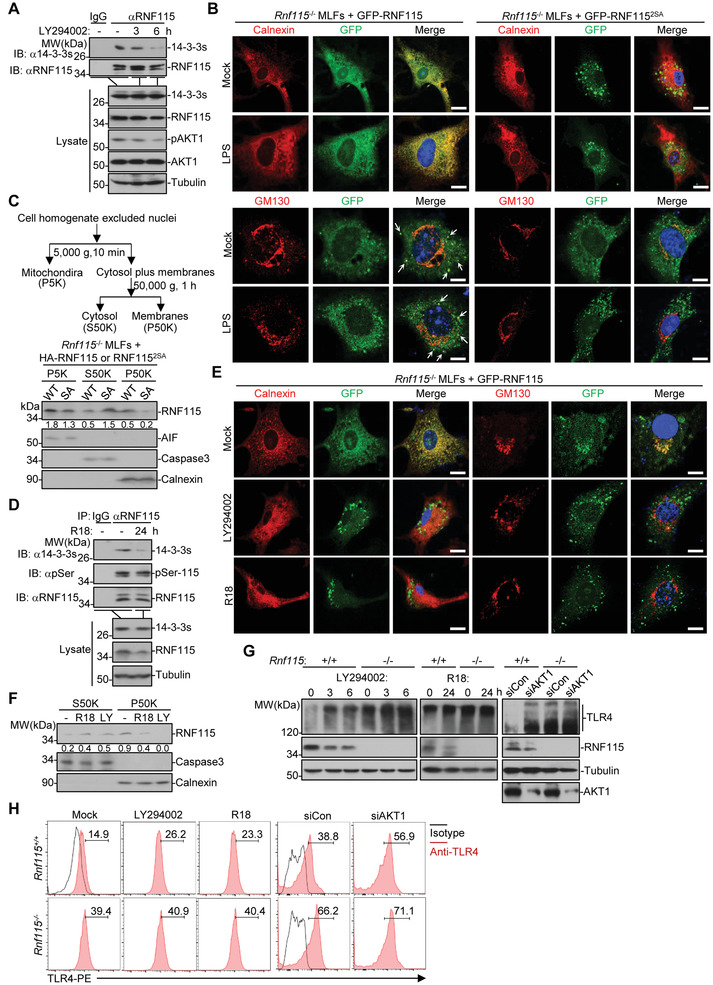
The 14‐3‐3 chaperones bind to AKT1‐phosphorylated RNF115 and facilitate RNF115 localizing on the ER and the Golgi apparatus. A) Immunoprecipitation (with IgG or anti‐RNF115) and immunoblot analysis (with anti‐14‐3‐3s, RNF115, p‐AKT1, AKT1, or Tubulin) of MLFs left untreated or treated with LY294002 (20 × 10^−6^ m) for 0, 3, or 6 h. B) Immunofluorescent staining Calnexin (red) or GM130 (red) and confocal microscopy imaging of *Rnf115* ^−/−^ MLFs reconstituted with GFP‐RNF115 or GFP‐RNF115^2SA^ that were left unstimulated or stimulated with LPS (1 µg mL^−1^) for 15 min. Arrows indicate the colocalization of RNF115 and GM130. C) *Rnf115* ^−/−^ MLFs stably transfected with HA‐RNF115 or HA‐RNF115^2SA^ followed by cell fractionation and immunoblot analysis (with anti‐RNF115, anti‐AIF, anti‐Caspase 3, or anti‐Calnexin). D) Immunoprecipitation (with IgG or anti‐RNF115) and immunoblot analysis (with anti‐14‐3‐3s, RNF115, p‐Ser, or Tubulin) of BMDCs left untreated or treated with R18 (10 × 10^−6^ m) for 24 h. E) Immunofluorescent staining Calnexin (red) or GM130 (red) and confocal microscopy imaging in *Rnf115* ^−/−^ MLFs reconstituted with GFP‐RNF115 treated with LY294002 (6 h) or R18 (24 h). F) Cell fractionation and immunoblot analysis of BMDCs untreated or treated with LY294002 (6 h) or R18 (24 h). G) Immunoblot analysis (with anti‐TLR4, anti‐RNF115, or antitubulin) in *Rnf115* ^+/+^ and *Rnf115* ^−/−^ BMDCs left untreated or treated with LY294002 (3–6 h) or R18 (24 h), or transfected with siCon or siAKT1 for 48 h. H) Flow cytometry analysis of cell surface TLR4 on *Rnf115* ^+/+^ and *Rnf115* ^−/−^ BMDCs left untreated or treated with LY294002 (6 h) or R18 (24 h), or transfected with siCon or siAKT1 for 48 h. Scale bars represent 5 µm. Data are representative of three independent experiments.

RNF115 lacks any recognizable transmembrane domains and has been implicated to localize on the ER.^[^ ^35^ ^]^ We thus performed unbiased mass spectrometry assays to identify potential chaperones that bind to RNF115 and facilitate the ER localization of RNF115. Several 14‐3‐3 chaperones were found as RNF115‐interacting proteins in mass spectrometry assays and in subsequent co‐immunoprecipitation assays (14‐3‐3 theta, eta, zeta, epsilon, gamma, and beta) (Table S1 and Figure S4A, Supporting Information). The 14‐3‐3 chaperones are known as phosphoserine/threonine‐binding proteins that transport the target proteins from the cytoplasm to the subcellular compartments.^[^ ^41^ ^]^ Interestingly, RNF115 contains an AKT phosphorylation site (Ser132/133) within the potential 14‐3‐3 binding domain.^[^ ^42^, ^43^ ^]^ We experimentally confirmed that RNF115 was phosphorylated in BMDCs in the presence or absence of LPS or Poly(I:C) treatment which was attenuated by the AKT1 inhibitor LY294002 (Figure S4B, Supporting Information). In addition, mutation of Ser132 and Ser133 residues into Ala residues (RNF115^2SA^) impaired phosphorylation of RNF115 (Figure S4C, Supporting Information). Knockdown of AKT1 but not AKT2 or AKT3 inhibited the phosphorylation of RNF115 (Figure S4D, Supporting Information), indicating that AKT1 is the main kinase for the phosphorylation of RNF115 at Ser132 and Ser 133. Interestingly, RNF115^2SA^ markedly lost the ability to associate with 14‐3‐3 chaperones compared to wild‐type RNF115 (Figure S4E, Supporting Information). Consistently, treatment with the AKT inhibitor LY294002 impaired the association between RNF115 and the 14‐3‐3 chaperones (**Figure** **4**A). These findings together indicate that AKT1‐mediated phosphorylation on Ser132 and Ser133 of RNF115 is required for the association between RNF115 and 14‐3‐3 chaperones.

### 2.7 The 14‐3‐3 Chaperones Facilitate RNF115 Localizing on the ER and the Golgi Apparatus

Cell fractionation assays and immunofluorescent confocal microscopy analyses suggested that RNF115^2SA^ lost its ability to localize on the ER and the Golgi apparatus compared to wild‐type RNF115 (Figure 4B,C). Expectedly, RNF115^2SA^ failed to interact with TLR4 and TLR9 in co‐immunoprecipitation assays and to downregulate the surface expression of TLR4 in flow cytometry analysis (Figure 4F; Figure S3F, Supporting Information). In addition, reconstitution of RNF115^2SA^ into *Rnf115* ^−/−^ MLFs failed to inhibit LPS‐ or poly(I:C)‐induced expression of proinflammatory cytokines and phosphorylation of IRF3, TBK1, or p38 as did wild‐type RNF115 (Figure S4G–I, Supporting Information). These data collectively suggest that the phosphorylation on Ser132/133 of RNF115 mediates the localization of RNF115 on the ER and the Golgi apparatus and is required for RNF115‐mediated downregulation of TLRs‐induced signaling.

R18 is a polypeptide that interferes with the target‐interacting interface of 14‐3‐3 chaperones.^[^ ^44^ ^]^ We found that treatment with R18 impaired the association between 14‐3‐3 chaperones and RNF115 without affecting the phosphorylation of RNF115 (Figure 4D). Consistently, treatment with LY294002 or R18 substantially inhibited the localization of RNF115 on the ER and the Golgi apparatus (Figure 4E,F). In addition, treatment with LY294002 or R18 or knockdown of AKT1 increased the glycosylation and the surface expression of TLR4 in *Rnf115* ^+/+^ BMDCs but had minimal effect on the glycosylation and the surface expression of TLR4 in *Rnf115* ^−/−^ BMDCs (Figure 4G,H), indicating RNF115 as a primary target of AKT1 and R18 for TLRs glycosylation and trafficking. These data together suggest that the 14‐3‐3 chaperones facilitate RNF115 localizing on the ER and the Golgi apparatus to inhibit TLRs trafficking and signaling.

### 2.8 RNF115 Interacts with RAB1A to Inhibit Post‐ER Trafficking of TLRs

**Figure 5:**
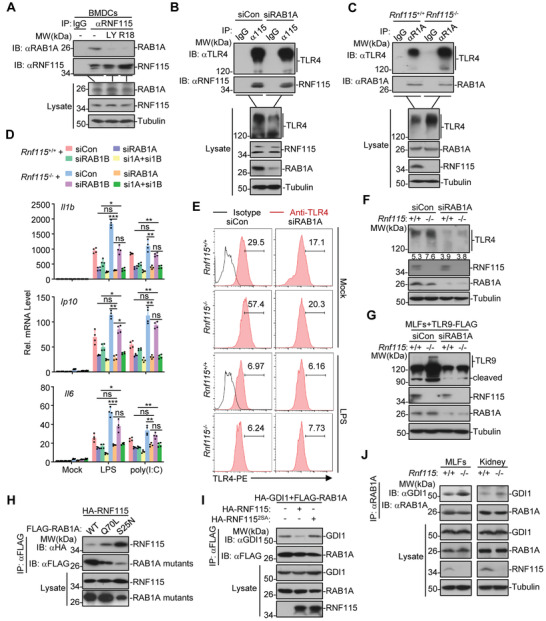
RNF115 interacts with RAB1 to inhibit the post‐ER trafficking of TLRs. A) Immunoprecipitation (with IgG or anti‐RNF115) and immunoblot analysis (with anti‐RAB1A, RNF115, or Tubulin) of BMDCs that were left untreated or treated with LY294002 (6 h) or R18 (24 h). B) Immunoprecipitation (with IgG or anti‐RNF115) and immunoblot analysis (with anti‐TLR4, RNF115, RAB1A, or Tubulin) of MLFs transfected with siCon or siRAB1A for 48 h. C) Immunoprecipitation (with IgG or anti‐RAB1A) and immunoblot analysis (with anti‐TLR4, RNF115, RAB1A, or Tubulin) of *Rnf115* ^+/+^ and *Rnf115* ^−/−^ BMDCs. D) qPCR analysis of *Ip10*, *Il‐1b*, or *Il6* mRNA in *Rnf115* ^+/+^ and *Rnf115* ^−/−^ BMDCs transfected with siCon, siRAB1A, or siRAB1B for 48 h followed by treatment with LPS (1 µg mL^−1^) or poly(I:C) (50 µg mL^−1^) for 0–3 h. E) Flow cytometry analysis of the cell surface TLR4 of *Rnf115* ^+/+^ and *Rnf115* ^−/−^ BMDCs transfected with siCon or siRAB1A for 48 h followed by treatment with LPS (1 µg mL^−1^) for 0–15 min. F) Immunoblot analysis (with anti‐TLR4, anti‐RAB1A, anti‐RNF115, or antitubulin) in *Rnf115* ^+/+^ and *Rnf115* ^−/−^ BMDCs transfected with siCon or siRAB1A for 48 h. G) Immunoblot analysis (with anti‐FLAG, anti‐RAB1A, anti‐RNF115, or antitubulin) in *Rnf115* ^+/+^ and *Rnf115* ^−/−^ MLFs reconstituted with FLAG‐TLR9 followed by transfection with siCon or siRAB1A for 48 h. H) Immunoprecipitation (with anti‐FLAG) and immunoblot analysis (with anti‐FLAG or anti‐HA) of HEK293 cells transfected with plasmids encoding HA‐RNF115, FLAG‐tagged RAB1A, or RAB1A mutants for 24 h. I) Immunoprecipitation (with anti‐FLAG) and immunoblot analysis (with anti‐FLAG, anti‐HA, or anti‐GDI1) of HEK293 cells transfected with plasmids encoding HA‐RNF115 or HA‐RNF115^2SA^ together with HA‐GDI1 and FLAG‐RAB1A for 24 h. J) Immunoprecipitation (with anti‐RAB1A) and immunoblot analysis (with anti‐RAB1A, RNF115, GDI1, or Tubulin) of the lysates from *Rnf115* ^+/+^ and *Rnf115* ^−/−^ MLFs or kidneys. *p < 0.05, **p < 0.01, ***p < 0.001 and ns (not significant) (two‐tailed Student's *t*‐test). Data are representative of three independent experiments (graphs show mean ± SD in (D)).

Having characterized that RNF115 is localized on the ER and the Golgi apparatus to inhibit the post‐ER trafficking of TLRs, we next examined the mechanisms by identifying the direct substrates of RNF115. The small GTPases RAB proteins mediate intracellular vesicles trafficking between different subcellular compartments.^[^ ^16^, ^45^, ^46^ ^]^ Interestingly, further analyses with the data from two independent mass spectrometry assays suggested RAB1A as an RNF115‐interacting protein which was confirmed by co‐immunoprecipitation assays (Table S1 and Figure S5A, Supporting Information). In addition, TLR4 and TLR9 are preferentially associated with RAB1A versus other RABs including RAB5A and RAB7A (Figure S5B, Supporting Information). The purified His‐RAB1A directly pulled down the in vitro generated RNF115 (Figure S5C, Supporting Information), indicating a direct association between RNF115 and RAB1A. Results from endogenous co‐immunoprecipitation assays suggested that RNF115 constitutively interacted with RAB1A in the presence or absence of LPS or poly(I:C) stimulation (Figure S5D, Supporting Information), and such association was substantially impaired by the treatment with LY294002 or R18 (**Figure** **5**A). Consistently, RNF115^2SA^ was not colocalized with or associated with RAB1A (Figure S5E,F, Supporting Information). These data together suggest that the phosphorylation and the ER localization of RNF115 are necessary for its association with RAB1A.

We next examined whether RAB1A mediates TLR trafficking and signaling downstream of RNF115. We found that knockdown of RAB1A did not affect the association between RNF115 and TLR4 (Figure 5B), whereas knockout of RNF115 enhanced the association between RAB1A and TLR4 (Figure 5C), indicating that RNF115 functions upstream of RAB1A to inhibit the association between RAB1A and TLR4. In addition, knockdown of RAB1A but not RAB1B (a homolog to RAB1A) significantly inhibited both LPS‐ and poly(I:C)‐induced expression of downstream genes in both *Rnf115* ^+/+^ BMDCs and *Rnf115* ^−/−^ BMDCs (Figure 5D; Figure S5G, Supporting Information). We further found that RNF115 preferentially interacted with RAB1A but not RAB1B in HEK293 cells (Figure S5H, Supporting Information). Consistently, knockdown of RAB1A downregulated the surface expression level and the glycosylation extent of TLR4 in *Rnf115* ^+/+^ BMDCs; these levels were similar to those in *Rnf115* ^−/−^ BMDCs (Figure 5E,F), suggesting that RAB1A is required for the glycosylation and the surface expression of TLR4 in both *Rnf115* ^+/+^ and *Rnf115* ^−/−^ BMDCs. Consistent with this notion, the majority of TLR4 was found to be localized on the ER in *Rnf115* ^+/+^ and *Rnf115* ^−/−^ MLFs after transfection of siRAB1A (Figure S5I, Supporting Information). Similarly, knockdown of RAB1A impaired the glycosylation and the cleavage of TLR9 (Figure 5G), and downregulated the localization of TLR9 on the Golgi apparatus and the endolysosomes in both *Rnf115* ^+/+^ MLFs and *Rnf115* ^−/−^ MLFs reconstituted with TLR9‐GFP in the presence or absence of CpG treatment (Figure S5J, Supporting Information). These data collectively suggest that RAB1A functions downstream of RNF115 for the post‐ER trafficking of TLRs.

RAB proteins are transformed between active [guanosine triphosphate (GTP)‐bound] and inactive [guanosine diphosphate (GDP)‐bound] forms for vesicle trafficking which is tightly controlled by a number of regulatory proteins such as GDP dissociation inhibitor (GDI), guanine nucleotide‐exchange factor (GEF), and GTPase activating protein (GAP).^[^ ^47^ ^]^ Specifically, the inactive GDP‐bound form of RAB1A is extracted from the vesicle membrane by the GDP dissociation inhibitor (GDI), which solubilizes the inactive RAB1A for GEF‐mediated activation of RAB1A during which the GDP‐bound RAB1A is transformed into GTP‐bound RAB1A for vesicular trafficking.^[^ ^48^ ^]^ Consistent with this notion, we found that the inactive mutant RAB1A(S25N) had a stronger association with GDI1 than wild‐type RAB1A (Figure S6A, Supporting Information). Interestingly, the inactive mutant RAB1A(S25N) associated with RNF115 more intensively than did wild‐type RAB1A (Figure 5H). In addition, wild‐type RNF115 but not RNF115^2SA^ substantially impaired the RAB1A‐GDI1 associations and the RAB1A(S25N)‐GDI1 associations (Figure 5I; Figure S6A, Supporting Information), indicating that RNF115 associates with the inactive RAB1A to inhibit its association with GDI. In support of this notion, knockout of RNF115 potentiated the RAB1A‐GDI1 associations in kidney or MLFs (Figure 5J). These data together indicate that RNF115 associates with RAB1A to inhibit the RAB1A‐GDI associations and the subsequent cycling of the active RAB1A from the inactive RAB1A.

### 2.9 RNF115 Catalyzes K11‐Linked Ubiquitination on Lys49 and Lys61 of RAB1A

**Figure 6:**
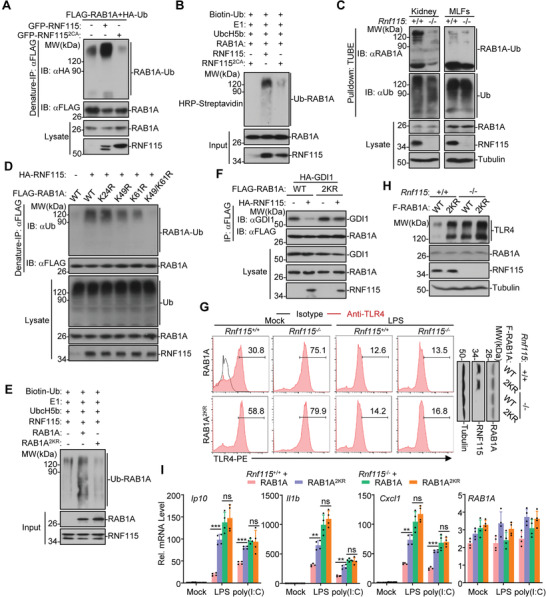
RNF115 catalyzes K11‐linked ubiquitination of RAB1 at Lys49/61. A) Denature‐IP (with anti‐FLAG) and immunoblot analysis (with anti‐FLAG, anti‐HA, or anti‐GFP) of HEK293 cells transfected with plasmids encoding FLAG‐RAB1A and HA‐Ubiquitin together with an empty vector, GFP‐RNF115, or GFP‐RNF115^2CA^ for 24 h. B) In vitro ubiquitination analysis of RAB1A by RNF115. RAB1A, RNF115, and RNF115^2CA^ were obtained by an in vitro transcription and translation kit. Ubiquitin‐conjugated RAB1A was detected by immunoblot with HRP‐streptavidin (upper panel). The proteins in the input were detected by immunoblot with the indicated antibodies (lower panels). C) Immunoprecipitation (with GST beads and GST‐TUBE) and immunoblot analysis (with anti‐RAB1A, anti‐Ub, anti‐RNF115, antitubulin) of the lysates from *Rnf115* ^+/+^ and *Rnf115* ^−/−^ MLFs or kidneys. D) Denature‐IP (with anti‐FLAG) and immunoblot analysis (with anti‐FLAG, anti‐HA or anti‐Ub) of HEK293 cells transfected with plasmids encoding HA‐RNF115 together with FLAG‐tagged RAB1A or RAB1A mutants for 24 h. E) In vitro ubiquitination analysis of RAB1A or RAB1A(K49/61R) (RAB1^2KR^) by RNF115. RAB1A, RAB1^2KR,^ and RNF115 were obtained by an in vitro transcription and translation kit. Ubiquitin‐conjugated RAB1A or RAB1A^2KR^ was detected by immunoblot with HRP‐streptavidin (upper panel). The proteins in the input were detected by immunoblot with the indicated antibodies (lower panels). F) Immunoprecipitation (with anti‐FLAG) and immunoblot analysis (with anti‐FLAG, anti‐HA, or anti‐GDI1) of HEK293 cells transfected with plasmids encoding HA‐RNF115, HA‐GDI1, FLAG‐RAB1A, or FLAG‐RAB1A^2KR^ for 24 h. G) Flow cytometry analysis of cell surface TLR4 on *Rnf115* ^+/+^ and *Rnf115* ^−/−^ MLFs reconstituted with FLAG‐RAB1A or RAB1A^2KR^ followed by treatment with LPS (1 µg mL^−1^) for 0–15 min. The expression levels of RAB1A and RAB1A^2KR^ were determined by immunoblot analysis. H) Immunoblot analysis (with anti‐TLR4, anti‐FLAG, anti‐RNF115, or antitubulin) in *Rnf115* ^+/+^ and *Rnf115* ^−/−^ MLFs reconstituted with FLAG‐RAB1A or RAB1A^2KR^. I) qPCR analysis of *Ip10, Il1b, Cxcl1*, or *RAB1A* mRNA in *Rnf115* ^+/+^ and *Rnf115* ^−/−^ MLFs reconstituted with FLAG‐RAB1A or RAB1A^2KR^ followed by treatment with LPS (1 µg mL^−1^) or poly(I:C) (50 µg mL^−1^) for 0–3 h. **p < 0.01, ***p < 0.001 and ns (not significant) (two‐tailed Student's *t*‐test). Data are representative of three independent experiments (graphs show mean ± SD in (H)).

Because RAB1A directly associates with and functions downstream of RNF115 for TLR trafficking and the enzymatic activity of RNF115 is required for its inhibition of TLR trafficking, we hypothesized that RNF115 might target RAB1A for ubiquitination. As shown in **Figure** **6**A,B and Figure S6B (Supporting Information), RNF115 catalyzed ubiquitination of RAB1A and RAB1A(S25N) in cells and in vitro. Consistently, the ubiquitination of RAB1A was impaired in *Rnf115* ^−/−^ kidney or MLFs compared to the *Rnf115* ^+/+^ counterparts (Figure 6C). In addition, treatment with LY294002 or R18 abolished RNF115‐mediated ubiquitination of RAB1A (Figure S6C, Supporting Information), which is in agreement with the observations that treatment with LY294002 or R18 impairs the RNF115‐RAB1A associations (Figure 5A). These data suggest that RNF115 catalyzes ubiquitination of RAB1A.

We next investigated the types of polyubiquitin chains of RAB1A that are mediated by RNF115. We cotransfected FLAG‐RAB1A with HA‐tagged ubiquitin, ubiquitin mutants retaining a single lysine residue (KO), or ubiquitin mutants with one of the seven lysine residues mutated into arginine (KR) into HEK293 cells in the presence or absence of RNF115 followed by ubiquitination assays. The results showed that knockdown of RNF115 markedly impaired the targeting of Ub(K11O) (a ubiquitin mutant in which all lysine residues but Lys11 were mutated into Arg) to RAB1A but had little effect on the targeting of Ub(K11R) (a ubiquitin mutant in which Lys11 was mutated into Arg) to RAB1A (Figure S6D,E, Supporting Information),^[^ ^49^ ^]^ suggesting that RNF115 catalyzes K11‐linked poly‐ubiquitination of RAB1A.

The switch I and switch II motifs of RAB1A constitute two interfaces associated with GDI.^[^ ^50^ ^]^ Sequence analysis identified two lysine residues (K49 and K61) flanking the two switch modules of RAB1A which were conserved in RAB1A from different species but not in RAB5A, RAB7A, RAB24, or RAB32 from the same species (Figure S7A,B, Supporting Information). Mutation of either Lys49 or Lys61 into Arg was partially and simultaneous mutation of these two residues of RAB1A into Arg was completely insensitive to RNF115‐mediated ubiquitination (Figure 6D,E; Figure S7C, Supporting Information). In contrast, mutation of the Lys24 residue of RAB1A into Arg which is conserved in the P‐loop of RAB proteins had no effect on RNF115‐mediated ubiquitination of RAB1A (Figure 6D; Figure S7C, Supporting Information). These data indicate that RNF115 catalyzes K11‐linked ubiquitination on Lys49 and Lys61 of RAB1A.

We next performed mass spectrometry (MS) assays to further confirm the ubiquitination on Lys49 and Lys61 of RAB1A. With trypsin as the digesting enzyme, however, one of the modified peptides would be “TIK(GG)” which might be too short to be detected by MS analysis. We thus mutated (K58T) a trypsin site in RAB1A (RAB1A^K58T^) to lengthen the target modified peptide. Importantly, RNF115 induced ubiquitination of RAB1A^K58T^ to a similar extent as that of wild‐type RAB1A (Figure S7D, Supporting Information), indicating that mutation of Lys58 into Thr has no effect on RNF115‐mediated ubiquitination of RAB1A. We next purified RAB1A^K58T^ from HEK293 cells that were transfected with FLAG‐RAB1A^K58T^ together with an empty control vector or RNF115 and performed in‐solution digestion and MS analysis ^[^ ^51^ ^]^(Figure S7E, Supporting Information). The results showed that the unmodified peptide “TIELDGTTIK” was identified when RAB1A^K58T^ was cotransfected with RNF115 or with the empty vector into HEK293 cells. However, the “TIELDGTTIK(GG)LQIWDTAGQER” peptide was only identified when RAB1A^K58T^ was cotransfected with RNF115 (Figure S7F, Supporting Information). These data demonstrate that the Lys61 residue of RAB1A is modified by ubiquitin in the presence of RNF115.

### 2.10 RAB1A^K49/61R^ Promotes TLRs Trafficking and TLRs‐Mediated Signaling

We further found that RAB1A(K49/61R) (RAB1A^2KR^) still interacted with GDI1 (Figure 6F). However, RNF115 did not impair the associations between RAB1A^2KR^ and GDI1 as it did for the associations between wild‐type RAB1A and GDI1 (Figure 6F), indicating that RNF115‐mediated ubiquitination of RAB1A inhibits the association between RAB1A and GDI. These observations together with the structural evidence suggest that RNF115 catalyzes K11‐linked ubiquitination on Lys49 and Lys61 of RAB1A to inhibit the RAB1A‐GDI interaction (Figure S8A, Supporting Information).

We next investigated whether and how RAB1A^2KR^ regulates TLRs trafficking and signaling. The *Rnf115* ^+/+^ and *Rnf115* ^−/−^ MLFs were stably transfected with RAB1A or RAB1A^2KR^ followed by various analyses. Results from flow cytometry and immunoblot analyses showed that RAB1A^2KR^ promoted the glycosylation and the surface expression of TLR4 more intensively than did wild‐type RAB1A in *Rnf115* ^+/+^ MLFs in the absence of LPS treatment (Figure 6G,H). In contrast, RAB1A^2KR^ slightly (if any) increased the glycosylation and the surface expression of TLR4 compared to wild‐type RAB1A in *Rnf115* ^−/−^MLFs without LPS treatment (Figure 6G,H). Treatment with LPS downregulated the surface expression of TLR4 to comparable levels in both *Rnf115* ^+/+^ and *Rnf115* ^−/−^ MLFs that were stably transfected with RAB1A or RAB1A^2KR^ (Figure 6G), indicating that RAB1A^2KR^ does not inhibit LPS‐induced endocytosis of TLR4 compared to wild‐type RAB1A. In addition, RAB1A^2KR^ restored the glycosylation of TLR4 and TLR9 and the cleavage of TLR9 that were downregulated by RNF115 (Figure S8B, Supporting Information), suggesting that RAB1A^2KR^ renders resistance to RNF115‐downregulated glycosylation and cleavage of TLRs. Consistent with this notion, RAB1A^2KR^ significantly increased both LPS‐ and poly(I:C)‐induced expression of downstream genes including *Ip10*, *Il1b*, and *Cxcl1* in *Rnf115* ^+/+^ MLFs compared to wild‐type RAB1A (Figure 6I). In contrast, LPS‐ and poly(I:C)‐induced expression of downstream genes was comparable between *Rnf115* ^−/−^ MLFs stably transfected with RAB1A^2KR^ and those with wild‐type RAB1A (Figure 6I). Taken together, these data collectively indicate that RNF115‐mediated ubiquitination on Lys49 and Lys61 of RAB1A inhibits the post‐ER trafficking of TLRs and TLRs‐mediated signaling.

### 2.11 RNF115 Catalyzes K11‐Linked Ubiquitination on Lys46 and Lys58 of RAB13

**Figure 7:**
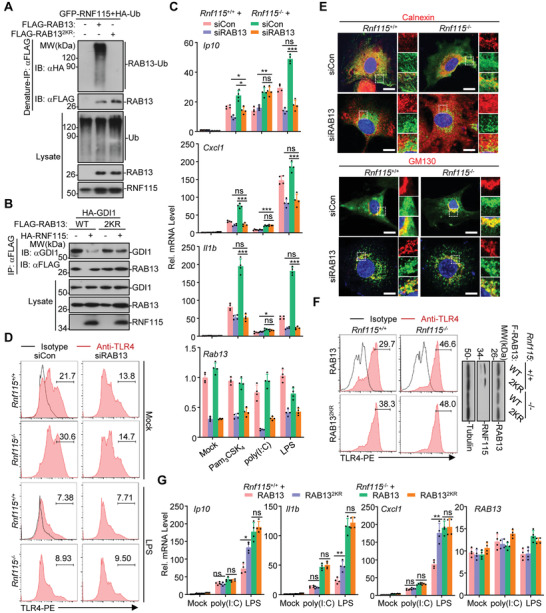
RNF115 catalyzes K11‐linked ubiquitination on Lys46 and Lys58 of RAB13 to inhibit the post‐Golgi trafficking of TLR4 and TLR9. A) Denature‐IP (with anti‐FLAG) and immunoblot analysis (with anti‐FLAG, anti‐Ub, or anti‐GFP) of HEK293 cells transfected with plasmids encoding FLAG‐RAB13 together with an empty vector, GFP‐RNF115 or GFP‐RNF115^2CA^ for 24 h. B) Immunoprecipitation (with anti‐FLAG) and immunoblot analysis (with anti‐FLAG, anti‐HA, or anti‐GDI1) of HEK293 cells transfected with plasmids encoding HA‐RNF115, HA‐GDI1, FLAG‐RAB13, or RAB1A(K46/58R) (RAB13^2KR^) for 24 h. C) qPCR analysis of *Ip10*, *Il1b*, *Cxcl1*, or *Rab13* mRNA in *Rnf115* ^+/+^ and *Rnf115* ^−/−^ BMDCs transfected with siCon, siRAB13 for 48 h followed by treatment with LPS (1 µg mL^−1^), Pam_3_CSK_4_ (1 µg mL^−1^) or poly(I:C) (50 µg mL^−1^) for 0–3 h. D) Flow cytometry analysis of the cell surface TLR4 of *Rnf115* ^+/+^ and *Rnf115* ^−/−^ BMDCs transfected with siCon or siRAB13 for 48 h followed by treatment with LPS (1 µg mL^−1^) for 0–15 min. E) Immunofluorescent staining Calnexin (red), GM130 (red), or TLR4 (green), and confocal microscopy analysis in *Rnf115* ^+/+^ and *Rnf115* ^−/−^ MLFs transfected with siCon or siRAB13 for 48 h. F) Flow cytometry analysis of cell surface TLR4 on *Rnf115* ^+/+^ and *Rnf115* ^−/−^ MLFs reconstituted with FLAG‐RAB13 or RAB13^2KR^. The expression levels of RAB13 and RAB13^2KR^ were determined by immunoblot analysis. G) qPCR analysis of *Ip10, Il1b, Cxcl1*, or *RAB13* mRNA in *Rnf115* ^+/+^ and *Rnf115* ^−/−^ MLFs reconstituted with FLAG‐RAB13 or RAB13^2KR^ followed by treatment with LPS (1 µg mL^−1^) or poly(I:C) (50 µg mL^−1^) for 0–3 h. *p < 0.05, **p < 0.01, ***p < 0.001 and ns (not significant) (two‐tailed Student's *t*‐test). Data are representative of three independent experiments (graphs show mean ± SD in (C) and (G)).

We have observed that RAB13 preferentially interacted with RNF115 versus RNF115^2SA^ (Figure S5F, Supporting Information). Interestingly, sequence alignment analysis suggested that the Lys46 and Lys58 residues of RAB13 exhibited homology with the Lys49 and Lys61 residues of RAB1A (Figure S9A, Supporting Information), which prompted us to speculate that RNF115 might catalyze the ubiquitination of RAB13 on these two sites and thereby inhibits the RAB13‐GDI interactions. As expected, RNF115 catalyzed K11O‐ but not K11R‐linked ubiquitination of RAB13 and inhibited the interactions between RAB13 and GDI1 (Figure S9B,C, Supporting Information). In addition, RNF115 neither catalyzed ubiquitination of RAB13(K46/58R) (RAB13^2KR^) nor impaired the interactions between RAB13^2KR^ and GDI1 (**Figure** **7**A,B). These data together suggest that RNF115 catalyzes K11‐linked ubiquitination on Lys46 and Lys58 of RAB13 to inhibit the GDI‐RAB13 interactions.

### 2.12 RAB13 Promotes the Post‐Golgi Trafficking of TLR4 and TLR9

RAB13 has been shown to regulate the trafficking of post‐Golgi vesicles to the recycling endosomes before being delivered to the plasma membrane.^[^ ^52^ ^]^ Interestingly, knockdown of RAB13 significantly inhibited Pam_3_CSK_4_‐, LPS‐, and CpG‐C‐induced expression of *Ip10*, *Cxcl1*, and *Il1b* in *Rnf115* ^+/+^ and *Rnf115* ^−/−^ BMDCs or pDCs (Figure 7C; Figure S9D, Supporting Information). In contrast, neither poly(I:C)‐ nor R848‐induced upregulation of *Ip10*, *Cxcl1*, and *Il1b* was affected by knockout of RNF115 in BMDCs or pDCs (Figure 7C; Figure S9D, Supporting Information). These data suggest that RAB13 is required for TLR2, TLR4, and TLR9 but not for TLR3 and TLR7 signaling.

We next examined whether RAB13 affected the localization of TLR4 and TLR9 in the presence or absence of ligands stimulation. As shown in Figure 7D, knockdown of RAB13 markedly downregulated the cell surface TLR4 but did not affect LPS‐induced downregulation of cell surface TLR4 in both *Rnf115* ^+/+^ and *Rnf115* ^−/−^ BMDCs. Results from immunofluorescent staining and confocal imaging analysis suggested that knockdown of RAB13 resulted in the formation of TLR4 puncta in cells and a portion of the TLR4 puncta was localized at the Golgi apparatus in *Rnf115* ^+/+^ and *Rnf115* ^−/−^ MLFs (Figure 7E), indicating that RAB13 is required for the proper post‐Golgi trafficking of TLR4 to the cell surface. Similarly, knockdown of RAB13 substantially inhibited the localization of TLR9 at endolysosomes after CpG stimulation in both *Rnf115* ^+/+^ and *Rnf115* ^−/−^ MLFs stably transfected with TLR9‐GFP (Figure S9E, Supporting Information). In contrast, the localization of TLR9 at the Golgi apparatus was not affected by knockdown of RAB13 in either *Rnf115* ^+/+^ or *Rnf115* ^−/−^ MLFs stably transfected with TLR9‐GFP (Figure S9E, Supporting Information). Together with the notion that TLR9 is transported from the Golgi apparatus to endolysosomes via the plasma membrane,^[^ ^22^ ^]^ these data suggest that RAB13 controls the post‐Golgi trafficking of TLR9 to the cell surface and then en route to the endolysosomes.

Because RNF115 catalyzed ubiquitination on Lys46 and Lys58 of RAB13 to inhibit the RAB13‐GDI interactions (Figure 7A,B), we next examined whether such ubiquitination inhibited the trafficking of TLRs. As shown in Figure 7f, RAB13^2KR^ substantially upregulated the surface TLR4 compared to wild‐type RAB13 when reconstituted into *Rnf115* ^+/+^ MLFs but not *Rnf115* ^−/−^ MLFs. Consistently, RAB13^2KR^ significantly upregulated LPS‐indued expression of *Ip10*, *Il1b*, and *Cxcl1* in *Rnf115* ^+/+^ MLFs but not *Rnf115* ^−/−^ MLFs compared to wild‐type RAB13 (Figure 7G). In contrast, RAB13^2KR^ did not upregulate poly(I:C)‐indued expression of *Ip10*, *Il1b*, and *Cxcl1* in either *Rnf115* ^+/+^ or *Rnf115* ^−/−^ MLFs compared to wild‐type RAB13 (Figure 7G), consistent with the observation that knockdown of RAB13 did not affect the expression of downstream genes in *Rnf115* ^+/+^ and *Rnf115* ^−/−^ BMDCs stimulated with poly(I:C) (Figure 7C). These data together indicate that RNF115 catalyzes K11‐linked ubiquitination on Lys46 and Lys58 of RAB13 to inhibit the post‐Golgi trafficking of TLR4 and TLR4‐mediated signaling.

## 3 Discussion

**Figure 8:**
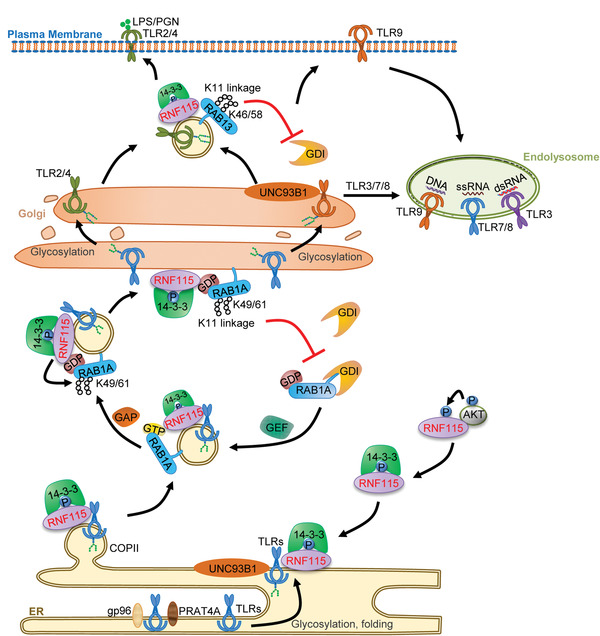
RNF115‐mediated step‐wise inhibition of the post‐ER trafficking of TLRs. The 14‐3‐3 chaperones bind to AKT1‐phosphorylated RNF115 and facilitate RNF115 localizing on the ER. RNF115 interacts with TLRs and buds off the ER onto the coat protein complex II vesicles. RAB1A is recruited to the TLR‐containing vesicles and promotes the trafficking of the vesicles to the Golgi apparatus. Simultaneously, the active form of RAB1A is transformed into the inactive form. RNF115 catalyzes K11‐linked ubiquitination on Lys49 and Lys61 of RAB1A to inhibit the extraction of RAB1A by GDI1. In addition, RNF115 inhibits the post‐Golgi trafficking of TLR2, TLR4, and TLR9 by catalyzing K11‐linked ubiquitination on Lys46 and Lys58 of RAB13. Similarly, RNF115‐mediated ubiquitination of RAB13 impairs its association with GDI. Consequently, the trafficking of TLR4 from the Golgi apparatus to cell surface and the trafficking of TLR9 to cell surface en route to endolysosomes were inhibited.

TLRs exit from the ER to their ultimate destinations in UNC93B1‐dependent and independent manners, which are essential for the proper posttranslational modifications, localization, signaling, and functioning of TLRs.^[^ ^15^, ^53^, ^54^ ^]^ The studies presented here have demonstrated RNF115 as a common regulator for post‐ER trafficking and signaling of UNC93B1‐dependent and independent TLRs (**Figure** **8**). Firstly, we found that overexpression of RNF115 impaired the Endo H‐resistant glycosylation of TLR4 and TLR9. Conversely, knockdown or knockout of RNF115 potentiated the glycosylation of TLR4 and TLR9 and the cleavage of TLR3 and TLR9. Knockout of RNF115 increased the localization of TLR9 on the Golgi apparatus and the endolysosomal compartments and promoted the localization of TLR2 and TLR4 on the cell surface. Consequently, knockout of RNF115 potentiated the signaling and immune responses mediated by UNC93B1‐dependent and independent TLRs. Secondly, we showed that RNF115 interacted with RAB1A and catalyzed K11‐linked polyubiquitin chains on Lys49 and Lys61 of RAB1A. Such a modification impaired the association between RAB1A and GDI, thereby inhibiting the cycling of inactive‐to‐active RAB1A for vesicle trafficking from the ER to the Golgi apparatus.^[^ ^55^ ^]^ There are 13 TLRs encoded by mouse genome, all of which bud off from the ER to the Golgi apparatus. Thus, it is expected that the trafficking of all TLRs from the ER to the Golgi apparatus is regulated by the RNF115‐RAB1A axis. In contrast, RNF115 did not interact with RAB1B, a homologue to RAB1A, which might be due to the hypervariable C termini of RAB1A and RAB1B. Consistently, knockdown of RAB1A but not RAB1B inhibited the glycosylation of TLRs and TLR‐mediated signaling and resulted in the retention of TLR4 on the ER in *Rnf115* ^+/+^ and *Rnf115* ^−/−^ cells. In addition, RAB1A^2KR^ potentiated the glycosylation and the cell surface expression of TLR4 in *Rnf115* ^+/+^ MLFs but not in *Rnf115* ^−/−^ MLFs, indicating RAB1A as a substrate of RNF115 to promote the post‐ER trafficking of TLRs and TLR‐mediated signaling. These findings provide direct evidence to support the notion that the ER exit of TLRs to the Golgi apparatus follows the traditional route of the conventional secretory pathway.^[^ ^16^, ^22^ ^]^

The conventional secretory pathway controls the exocytosis of a large number of proinflammatory cytokines and chemokines and the surface expression of transmembrane receptors.^[^ ^56^, ^57^, ^58^, ^59^, ^60^ ^]^ Interestingly, however, we found that neither TNF‐ nor IL‐1*β*‐induced secretion of CXCL1 or IL‐6 was affected by knockout of RNF115. In addition, the cell surface expression of MHC‐I and the integrin molecules such as CD11b or CD11c which is mediated by the conventional secretory pathway was comparable between *Rnf115* ^+/+^ and *Rnf115* ^−/−^ BMDCs. These data indicate that RNF115‐mediated ubiquitination of RAB1A and RAB13 selectively inhibits the post‐ER trafficking of TLRs‐containing vesicles. Although how RNF115 is specified for TLR trafficking via the conventional secretory pathway is unclear, it possibly involves additional regulatory proteins that bridge TLRs and RNF115 on the ER, which requires further investigations.

We further found that RNF115 catalyzed K11‐linked polyubiquitin chains on Lys46 and Lys58 of RAB13 (homology to Lys49 and Lys61 of RAB1A). Such a modification impaired the association between RAB13 and GDI, thereby inhibiting the trafficking of a subset of TLRs from the Golgi apparatus to the cell surface. Consistent with this notion, knockdown of RAB13 impaired the surface expression of TLR4 and the endolysosomal localization of TLR9 in both *Rnf115* ^+/+^ and *Rnf115* ^−/−^ MLFs. In addition, RAB13^2KR^ potentiated the cell surface expression of TLR4 in *Rnf115* ^+/+^ MLFs but not in *Rnf115* ^−/−^ MLFs, indicating that RNF115 inhibits the post‐Golgi trafficking of TLR4 and TLR9 by catalyzing ubiquitination on Lys46 and Lys58 of RAB13. Interestingly, however, knockdown of RAB13 did not inhibit TLR3‐ and TLR7‐mediated signaling in BMDCs and pDCs, respectively. It has been well recognized that TLR2/4/5/11/13 (TLR1 and TLR6 associate with TLR2 on cell membrane for signaling) are transported from the Golgi apparatus to cell membrane and TLR9 trafficks from the Golgi apparatus to cell membrane en route to endolysosome, whereas TLR3/7 traffick from the Golgi apparatus to endolysosome.^[^ ^22^ ^]^ It is thus likely that RAB13 mediates the trafficking of TLR2/4/5/9/11/13 from the Golgi apparatus to cell membrane, but does not mediate TLR3/7 trafficking from the Golgi apparatus to endolysosomes. Collectively, these data suggest that RAB1A and RAB13 regulate TLRs trafficking in a step‐wise manner, i.e., RAB1A mediates TLRs trafficking from the ER to the Golgi apparatus, whereas RAB13 mediates selective TLRs trafficking from the Golgi apparatus to the cell surface.

The lysine residues homologous to Lys49 and Lys61 of RAB1A have been identified in multiple RAB proteins, including RAB8A, RAB10, RAB18, RAB35, and RAB40A. It is thus conceivable that RNF115 also catalyzes the ubiquitination of these RAB proteins on the conserved lysine residues and thereby inhibits the subsequent recruitment of GDI. In addition, these RAB proteins extensively regulate the trafficking of intracellular vesicles to endosomes and plasma membranes in different types of cells or under distinct physiological and pathological conditions, in which it would be expected that RNF115 plays essential roles. In this context, it is of great interest to examine whether RNF115 regulates the post‐Golgi trafficking of TLR7 and TLR3 to endolysosomes by catalyzing the ubiquitination of the conserved lysine residues of these RAB proteins.

Previous and our current studies have shown that RNF115 interacts with RAB5A and RAB7A, both of which cooperatively regulate the phagocytosis and the endocytosis.^[^ ^61^ ^]^ Accordingly, RNF115 has been shown to promote EGFR endocytic trafficking and lysosomal degradation.^[^ ^32^, ^34^ ^]^ In our study, we found that AKT1‐mediated phosphorylation on Ser132 and Ser133 of RNF115 was required for the RNF115‐RAB1A associations. Consistently, RNF115^2SA^ failed to inhibit the glycosylation and the cleavage of TLRs and the expression of proinflammatory cytokines in *Rnf115* ^−/−^ MLFs after treatment with LPS and poly(I:C), suggesting that RNF115 negatively regulates TLR‐induced signaling in a manner dependent on the AKT1‐mediated phosphorylation of RNF115. However, RNF115^2SA^ still interacted with RAB5A and RAB7A, indicating that the phosphorylation on Ser132 and Ser133 of RNF115 is optional for its association with RAB5A and RAB7A. Thus, it is unlikely that RNF115 regulates the endocytic trafficking of TLRs by targeting RAB5A or RAB7A. In support of this notion, we observed that LPS‐induced downregulation of the cell surface TLR4 and Pam_3_CSK_4_‐induced downregulation of the cell surface TLR2 were comparable between *Rnf115* ^+/+^ and *Rnf115* ^−/−^ BMDCs. Neither knockdown of RAB1A or RAB13 nor reconstitution of RAB1A^2KR^ or RAB13^2KR^ in cells inhibited the downregulation of the cell surface TLR4 after treatment with LPS, indicating that the RNF115‐RAB axis does not inhibit the endocytotic trafficking of surface TLRs. In this context, a recent study has shown that ablation of RNF115 has no effect on the phagocytic uptake of particles in macrophages.^[^ ^62^ ^]^ Besides mediating endocytotic trafficking of vesicles, RAB7 has been shown to regulate mitochondria‐lysosome contacts.^[^ ^63^ ^]^ Interestingly, our study showed that both RNF115 and RNF115^2SA^ interacted with RAB7A and RNF115 was found in the mitochondria fractions.^[^ ^35^ ^]^ Further investigations are required to examine the ER‐independent and RAB‐dependent roles of RNF115 in the future.

TLRs play crucial roles in antimicrobial immunity, autoimmunity, tumorigenesis, and tumor metastasis.^[^ ^16^, ^64^, ^65^ ^]^ We found that RNF115 negatively regulated TLRs‐mediated autoimmunity and antimicrobial immunity. While accumulating evidence shows that RNF115 promotes cancer development and indicates RNF115 as a therapeutic target for cancer,^[^ ^30^, ^42^ ^]^ the identification of RNF115 as an inhibitor of the trafficking and the activation of TLRs raises the caution for autoimmunity when targeting RNF115 for cancer treatment. AKT1‐mediated phosphorylation of RNF115 and the subsequent binding to 14‐3‐3 proteins were essential for the ER localization of RNF115 and for the post‐ER trafficking of TLRs. In addition, RNF115^2SA^ that failed to be localized on the ER and Golgi apparatus lost the activity to inhibit TLR trafficking and signaling, indicating that the ER and Golgi localization of RNF115 is required for its inhibitory role of TLR trafficking. We have previously shown that RNF115 promotes the K63‐linked ubiquitination of the ER adaptor protein MITA and the activation of innate immune signaling against DNA viruses.^[^ ^35^ ^]^ It is possible that the ER localization of RNF115 also plays a role in its regulation of MITA‐mediated antiviral and autoimmune signaling. Our findings highlight RNF115‐mediated regulation of distinct cellular processes may play different roles in antimicrobial and autoimmune disease and defining the mechanisms underlying this regulation would help explain the etiology of certain infectious and autoimmune diseases.

## 4 Experimental Section

### Mice

The *Rnf115* ^−/−^ mice and the genotyping methods were previously described.^[^ ^35^ ^]^ All the animal experiments were approved by the animal care and use committe of Wuhan University (Approval NO. 21020A).

### IMQ‐Induced Psoriasis‐Like Mouse Model

Back skin of female *Rnf115* ^+/+^ and *Rnf115* ^−/−^ mice (8–10 weeks of age) was shaved and the remaining hair was removed using a depilatory cream. The shaved back skin was treated with a daily topical dose of 62.5 mg IMQ cream (5%) for 6 consecutive days. For skin inflammation, an objective scoring system based on the clinical PASI score for psoriasis patients was adopted to evaluate the severity of back skin inflammation in the IMQ‐induced psoriasis‐like mouse model. Specifically, erythema, scaling, and thickening was scored independently on a scale of 0 to 4: 0, none; 1, slight; 2, moderate; 3, marked; 4, very marked. The total score was obtained by adding the 3 index scores (score of 0–12).

### Mouse Infection with Salmonellaenterica typhimurium

*Salmonellaenterica typhimurium* (SL1344) (ST) were kindly provided by Dr. Shan Li (Huazhong Agricultural University). The age‐ and sex‐matched *Rnf115* ^+/+^ and *Rnf115* ^−/−^ mice were injected by gavage with approximately 1 × 10^7^ colony‐forming units (CFU) of *S. typhimurium* suspended in 200 µL PBS. The weight and survival of mice were monitored daily. To determine the bacterial counts, mice were killed 5 days post‐infection. Feces and tissue samples, including livers and spleens, were removed, weighed and homogenized for bacterial quantification. The number of CFUs in the homogenates was determined by plating the serial dilutions on agar plates.

### Keyhole Limpet Hemocyanin Immunization

For keyhole limpet hemocyanin (KLH) experiments, *Rnf115* ^+/+^ and *Rnf115* ^−/−^ mice were subcutaneously immunized with KLH (0.15 mg per mouse) emulsified in IFA in the presence of CpG‐C (1 µg g^−1^ body weight). Seven days after immunization, the mice were sacrificed for subsequent analyses. Cells from the spleen (3 × 10^6^) or draining lymph nodes (1 × 10^6^) were seeded in 24‐well plates and stimulated with KLH (5 mg mL^−1^) for 24 h. The cell culture supernatants were collected and analyzed for IFN‐*γ* concentration by ELISA. Germinal center (GC) B cells were determined by staining with CD19, GL7, and FAS. In addition, the sera of immunized mice were collected to measure anti‐KLH‐specific IgG by ELISA. In brief, 96‐well plates were precoated with KLH (5 mg mL^−1^) overnight and then blocked with PBS containing 10% FBS. The plates were washed and overlaid with serially diluted sera for 2 h at room temperature. After washing, the KLH‐specific Abs were determined with rat antimouse IgG conjugated with HRP. After further washing, the plates were stained using the tetramethylbenzidine substrate. The reaction was stopped with 1 M H_2_SO_4_, and the absorbance was measured with spectramax i3x (Molecular Devices).

### Administration of LPS, R848, and CpG‐B

Age‐ and sex‐matched *Rnf115* ^+/+^ and *Rnf115* ^−/−^ mice were grouped for the experiments. The mice were injected intraperitoneally with LPS (10 µg g^−1^ body weight), or injected intravenously with R848 (1 µg g^−1^ body weight) and CpG‐B (1 µg g^−1^ body weight). The survival status of the LPS‐injected mice was monitored at half an hour intervals. The blood cells and sera were collected at 2 h after the injection of R848 and CpG‐B for subsequent qPCR and ELISA analysis.

### Reagents, Antibodies, and Constructs

Poly(I:C) was used as described previously.^[^ ^35^ ^]^ CpG‐B (ODN1826) and CpG‐C (ODN 2395) were synthesized by Synbio Technologies. R18 peptide was synthesized from GenScript. LPS was purchased from Sigma. LY294002 was purchased from TOPSCIENCE. R848, Pam_3_CSK_4_, and PGN were purchased from invivogen. Recombinant mouse IFN‐*α*, IFN‐*β*, and TNF‐*α* was purchased from PeproTech. Recombinant mouse IL‐1*β* was purchased from CHAMOT Biotechnology. Immuno‐reagents were obtained as following: Mouse control IgG (Santa Cruz Biotechnology, sc‐2025); rabbit control IgG (Millipore,12‐370); HRP‐conjugated goat‐anti mouse or rabbit IgG (Thermo Scientific, PA1‐86717 and SA1‐9510); HRP‐conjugated mouse anti‐FLAG (Sigma, A8592); HRP‐conjugated mouse anti‐HA (ABclonal, AE025); mouse anti‐FLAG (Sungene, KM8002); anti‐GFP (ABclonal, AE012); antitubulin (KM9003); anti‐HA (Sigma, H6908); anti‐Ubiquitin (Santa Cruz Biotechnology, sc‐8017); anti‐p‐p65 (Cell Singling Technologies, 3033S); anti‐p‐p38 (Cell Singling Technologies, 4511); anti‐p38 (Cell Singling Technologies, 8690); anti‐p65 (sc‐8008); anti‐TBK1 (Abcam, 96328‐11); anti‐p‐TBK1 (Abcam, 109272), anti‐IRF3 (Santa Cruz Biotechnology, sc‐9082); anti‐p‐IRF3 (Cell Singling Technologies, 4947S); anti‐AIF(Santa Cruz Biotechnology, sc‐13116); anti‐Caspase 3(Cell Singling Technologies, 9662S); anti‐Calnexin (Abcam, ab22595); anti‐RNF115 (Abcam,187642); anti‐RAB1A (Cell Singling Technologies, 13075; Santa Cruz Biotechnology, sc‐377201); anti‐14‐3‐3 (Cell Singling Technologies, 95422); anti‐GDI1 (ABclonal, A5462); anti‐TLR4 (Cell Singling Technologies, 14358S); anti‐TLR4 (ABclonal, A5258); anti‐TLR2 (ABclonal, A11225); anti‐AKT1 (Cell Singling Technologies, 75692S); anti‐AKT2 (ABclonal, A18019); anti‐AKT3 (ABclonal, A12909); anti‐p‐AKT1 (Cell Singling Technologies, 9018S); anti‐p‐Ser/Thr (Abcam, ab17464); anti‐Na/K^+^ ATPase (Abcam, ab76020). The mammalian expression plasmids for RAB1A, RAB5A, RAB7A, RAB13, RAB24, RAB32, RAB1A (Q70L), RAB1A (S25N), and GDI1 were kindly provided by Dr. Shan Li (Huazhong Agricultural University).^[^ ^66^ ^]^ Mammalian expression plasmids for phage‐TLR4‐GFP, phage‐TLR9‐GFP, phage‐TLR4‐HA, phage‐TLR4‐FLAG, phage‐TLR9‐FLAG, phage‐TLR3‐FLAG, phage‐RAB1B‐FLAG, phage‐RAB1A‐FLAG and RAB1A mutants, RAB13‐FLAG and RAB13 mutants, 14‐3‐3s, RNF115, and RNF115 mutants and truncations were constructed by standard molecular biology techniques.

### Co‐Immunoprecipitation and Immunoblot Assays

These experiments were performed as previously described.^[^ ^35^, ^67^ ^]^ Cells were collected and lysed for 10 min with 700 µL lysis buffer (20 × 10^−3^ M Tris HCl, pH 7.4–7.5, 150 × 10^−3^ M NaCl, 1 × 10^−3^ M EDTA, 1% Nonidet P‐40) containing inhibitors for protease and phosphatases (APExBIO). Cell lysates (600 µL) were incubated with a control IgG or specific antibodies and protein G agarose for 2–4 h. The immunoprecipitates were washed three times by 1 mL prelysis buffer and subject to immunoblot analysis. The rest of lysates (100 µL) were subject to immunoblot analysis to detect the expression of target proteins.

### Protein Purification and Ni‐NTA Pull‐Down Assay

The pET28a‐6His‐RAB1A plasmid was transformed into BL21 competent cells which were induced with IPTG (0.1 × 10^−3^ M) at 18 °C for 16 h. The cells were suspended in PBS and broken by Ultrasonic cell crusher. The supernatants were filtered and subject to Nickel affinity chromatography column balanced by binding buffer (150 × 10^−3^ M NaCl, 20 × 10^−3^ M Tris, pH 8.0). The proteins bound to Ni^2+^ column were eluted with different concentration gradients of binding buffer containing imidazole (100 × 10^−3^–500 × 10^−3^ M). FLAG‐RNF115 proteins were eluted by FLAG peptide from the anti‐FLAG precipitates of HEK293 cells that were transfected with FLAG‐RNF115. His‐RAB1A proteins (0, 0.5, 1, or 2 µg) were incubated with FLAG‐RNF115 at 4 °C overnight followed by Ni‐NTA Pull‐Down for 2 h in PBS containing protease inhibitors. The Ni‐NTA agarose was washed three times with PBS and subject to immunoblot analysis.

### Ubiquitination Assays

For in vivo ubiquitination experiments, cells were lysed in regular lysis buffer (100 µL) and the cell lysates were denatured at 95 °C for 15 min in the presence of 1% SDS. A portion of cell lysates (15 µL) were saved for immunoblot analysis to detect the expression of target proteins. The rest of the cell lysates (85 µL) were diluted with 1 mL lysis buffer and immunoprecipitated (Denature‐IP) with anti‐FLAG (M2) antibody‐conjugated beads. The immunoprecipitates were washed three times and subject to immunoblot analysis.

For in vitro ubiquitination experiments, RNF115 and mutants were expressed with TNT Quick Coupled Transcription/Translation Systems kit (Promega, Madison, WI) as the manufacturer's instructions. Ubiquitination was analyzed with a ubiquitination kit (Enzo Life Sciences, Farmingdale, NY) following the protocols recommended by the manufacturer.

### Cell Fractionation Assays

The cell fractionation assay was performed as previously described.^[^ ^35^ ^]^ In brief, the MLF cells (6 × 10^7^) treated with R18 and LY294002 or left untreated were washed with PBS followed by dousing 20 times in 1 mL homogenization buffer (10 × 10^−3^ M Tris‐HCl [pH 7.4], 2 × 10^−3^ M MgCl2, 10 × 10^−3^ M KCl, and 250 × 10^−3^ M sucrose) by 1ml injector. The homogenate was centrifuged at 500g for 10 min. The supernatant (S5) was centrifuged at 5 000g for 10 min to precipitate the mitochondria (P5K). The supernatant from this step (S5K) was further centrifuged at 50 000 g for 60 min to yield P50K, which contains the membrane fraction, and S50K, which mainly consists of cytosol.

### qRT‐PCR and ELISA

Total RNA was extracted from cells using TRIzol (Life Technologies), and the first‐strand cDNA was reversed‐transcribed with All‐in‐One cDNA Synthesis SuperMix (Aidlab Biotechnologies). Gene expression was examined with a Bio‐Rad CFX Connect system by a fast two‐step amplification program with 2 x SYBR Green Fast qPCR Master Mix (Aidlab Biotechnologies). The value obtained for each gene was normalized to that of the gene encoding *β*‐actin. The primers for qPCR assays are listed in Table S2 in the Supporting Information. The IFN‐*α* (Biolegend), IFN‐*β* (Biolegend), IFN‐*γ* (Biolegend), IL‐6 (Biolegend), TNF (Elabscience), CXCL1 (4A Biotech), and CCL5 (4A Biotech) proteins in the sera or cell supernatants were determined by ELISA kits from the indicated manufacturers.

### Cell Culture

Bone marrow cells were isolated from femurs of *Rnf115* ^+/+^ and *Rnf115* ^−/−^ mice. The cells were cultured in DMEM containing 15% (vol/vol) FBS, 1% streptomycin‐penicillin. GM‐CSF(20 ng mL^−1^) and M‐CSF(10 ng mL^−1^) were added to the bone marrow culture for differentiation of BMDCs and BMDMs, respectively. For preparation of pDCs, bone marrow cells were cultured in RPMI medium containing 10% FBS, 1% streptomycin‐penicillin, and recombinant mouse Flt3L (40 ng mL^−1^, Peprotech). The medium was changed every 3 days. On day 7, cells were stained with CD11c, CD11b, and B220 followed by FACS sorting. The CD11c^+^CD11b^−^B220^+^ cells were collected as pDCs for subsequent analysis. HEK293 cells were from the American Type Culture Collection, authenticated by STR locus analysis, and tested for mycoplasma contamination. Primary MLFs were isolated from ∼8–12‐week‐old mice. Lungs were minced and digested in calcium and magnesium‐free HBSS buffer supplemented with 10 mg mL^−1^ type I collagenase (Worthington) and 20 µg mL^−1^ DNase I (Sigma‐Aldrich) for 3 h at 37 °C. Cell suspensions were cultured in DMEM containing 15% (vol/vol) FBS, 1% streptomycin‐penicillin. Two days later, adherent fibroblasts were rinsed with PBS and cultured for experiments.

### Lentivirus‐Mediated Gene Transfer

HEK293 cells were transfected with phage‐6tag‐RNF115, phage‐6tag‐RNF115^2CA^, phage‐6tag‐RNF115^2SA^, phage‐TLR9‐GFP, phage‐TLR9‐FLAG, phage‐TLR4‐GFP, phage‐RNF115‐GFP, phage‐RNF115^2SA^‐GFP, phage‐6tag‐RAB1A, phage‐6tag‐RAB1A^2KR^, phage‐6tag‐RAB13, phage‐6tag‐RAB13^2KR^ or the empty vector along with the packaging vectors psPAX2 and pMD2G. The medium was changed with fresh full medium (15% FBS, 1% streptomycin‐penicillin) at 4 h after transfection. Forty‐four hours later, the supernatants were harvested to infect MLFs in the presence of polybrene (4 µg mL^−1^) followed by various analyses.

### shRNA and siRNA

The shRNAs targeting RNF115 were constructed by plasmid pLentiLox 3.7 and transfected by Ultra Fection2.0 (4A Biotech) or transferred by lentivirus into cells followed by qPCR or Immunoblot analysis. The shRNA sequences used in this study are as follows: 5’‐GGTTTAGAGTGTCCAGTATGC‐3 ’.

The siRNAs targeting mouse RABs and AKTs were synthesized from GenePharma. Control siRNA: 5′‐UUCUCCGAACGUGUCACGUTT‐3′; siRAB1A: 5′‐GGAGUCCUUCAAUAACGUUTT‐3′; siRAB1B: 5′‐CCAGUGAGAAUGUCAAUAATT‐3′. siRAB13: 5′‐GCACUUACAUCUCUACCAUTT‐3′. siAKT1: 5′‐GCACCUUUAUUGGCUACAATT‐3′. siAKT2: 5′‐GCCGCUAUUAUGCCAUGAATT‐3′.siAKT3: 5′‐GCUCAUUCAUAGGCUAUAATT‐3′.

### Hematoxylin–Eosin Staining

The experiments were performed as previously described.^[^ ^68^ ^]^ Tissues from mice were fixed in 4% paraformaldehyde and embedded in paraffin blocks. The paraffin blocks were sectioned (5 µm) for HE staining (Beyotime Biotech) followed by coverslipped. Images were acquired using an Aperio VERSA 8 (Leica) multifunctional scanner.

### Immunofluorescence and Confocal Microscopy

MLFs were cultured on coverslips and fixed in 4% paraformaldehyde for 15 min and washed with PBS three times. After that, cells were permeabilized with 0.5% saponin in PBS for 5 min. After three washed in PBS, cells were blocked in 1% BSA, 0.1% saponin in PBS for 1 h. Slides were stained in blocking buffer with primary antibodies for 1 h followed by PBS wash three times. The cells were further stained with Alexa Fluor 488‐ or 594‐conjugated secondary antibodies for 1 h. Finally, cells were stained with the In Situ microplate nuclear stain and anti‐fade (DUO82064‐1KT) and covered onto slides. Images were acquired on an Olympus FV1000 fluorescence microscope.

### Surface Biotinylation Assay

MLFs were washed three times with ice‐cold PBS (pH 8.0) supplemented with 1 × 10^−3^ M MgCl_2_ and 2.5 × 10^−3^ M CaCl_2_. Then Sulfo‐NHS‐LC‐Biotin (Thermo Scientific, Waltham, MA, USA) was added to the same solution at 0.25 mg mL^−1^ and incubated with cells at 4 °C for 30 min with gentle rocking. The unbound biotin group was quenched by the addition of 0.1 M glycine. Total proteins were extracted with lysis buffer and incubated overnight at 4 °C with NeutrAvidin agarose beads (Thermo Scientific). The beads were washed three times with PBS (pH 8.0) and bound proteins were eluted with the boiling SDS sample buffer and used for subsequent immunoblot analysis.

### Deglycosylation Assay

Cells were lysed in lysis buffer (20 × 10^−3^ M Tris HCl, pH 7.4–7.5, 150 × 10^−3^ M NaCl, 1 × 10^−3^ M EDTA, 1% Nonidet P‐40) containing inhibitors for protease and phosphatases. Lysates were incubated with anti‐FLAG (M2) antibody‐conjugated beads at 4 °C for 4 h. Proteins were eluted and treated with Endo H or N‐glycosidase according to the manufacturer's instructions (New England Biolabs, Inc.). Samples were subjected to SDS‐PAGE and immunoblotting with FLAG antibodies.

### Flow Cytometry

Cells were re‐suspended in FACS buffer (PBS, 1% BSA) and stained with antibodies against surface markers. For intracellular TLR2 and TLR4 staining, cells were fixed and permeabilized by using a fixation and permeabilization solution followed by intracellular staining (BioLegend). Flow cytometry data were acquired on a FACSCelesta or Fortesa flow cytometer and analyzed with FlowJo software (TreeStar).

### LC‐MS Analysis

HeLa cells stable expressed FLAG‐RNF115 were untreated or treated with LPS or poly(I:C). Cells were subsequently prepared for co‐immunoprecipitation assays with anti‐FLAG affinity gel (M2 beads). The immunoprecipitates were washed three times by 1 mL prelysis buffer and eluted by FLAG peptides. The elutions were subject to LC‐MS analysis. For another attempt preparation for LC‐MS analysis, HeLa cells stably expressed Strep‐RNF115 or vector were collected. Strep‐RNF115 was purified by Strep‐Tactin® XT Starter Kit (IBA Lifesciences, Cat.No:2‐4998‐000) following the protocols recommended by the manufacturer. The purified proteins of two independent experiments were digested with trypsin overnight. The resulting peptide mixtures were desalted on SDB‐RPS stage tips and analyzed on the EASY‐nLC 1200 system interfaced online with the Orbitrap Exploris 480 mass spectrometer (Thermo Scientific) in DDA mode. Peptides were dissolved in 0.1% formic acid, loaded onto a C18 trap column (100 µm × 20 mm, 3 µm particle size, 120 Å pore size) through auto‐sampler and then eluted into a C18 analytical column (75 µm × 250 mm, 2 µm particle size, 100 Å pore size). Mobile phase A (0.1% formic acid) and mobile phase B (90% ACN, 0.1% formic acid) were used to establish a 60 min separation gradient. A constant flow rate was set at 300 nL min^−1^. Data was acquired using a spray voltage of 2 kV, Ion funnel RF of 40, and ion transfer tube temperature of 320 °C. Each scan cycle consisted of one full‐scan mass spectrum (Res. 60K, scan range 350 – 1500 m/z, AGC 300%, IT 20 ms) followed by MS/MS events (Res. 15K, AGC 100%, IT auto). Cycle time was set to 2 s. The isolation window was set at 1.6 Da. Dynamic exclusion time was set to 35 s. The normalized collision energy was set at 30%. The MS data were analyzed by Proteome Discoverer (Thermo Scientific, version 2.4).

To identify the ubiquitin‐modified lysine residues of RAB1A, the EASY‐nLC 1200 system interfaced online with the Orbitrap Exploris 480 mass spectrometer was adopted. In brief, FLAG‐tagged RAB1A^K58T^ together with RNF115 or an empty vector was transfected into HEK293 cells. Twenty‐four hours later, the FLAG‐RAB1A^K58T^ was immunoprecipitated with anti‐FLAG gel and eluted with 3xFLAG peptide (0.3 mg mL^−1^) in 1% SDC+ buffer (10 × 10^−3^ M TCEP, 40 × 10^−3^ M CAA, 1%SDC, 100 × 10^−3^ M Tris‐HCl). The elutions were collected and diluted with an equal volume of water to reduce the SDC concentration to 0.5%. The trypsin (1 µg) was added to the diluted elutions followed by overnight incubation at 37 °C while shaking. The reactions were quenched by TFA (fina concentration, 1%) followed by centrifuge for 5 min at 12 000g. The supernantants containing peptides were transferred to a fresh tube and desalted by SDB‐RPS stage tips. The desalted peptides were dissolved in MS loading buffer (0.1% formic acid), loaded onto a C18 trap column (100 µm x 20 mm, 3 µm particle size, 120 Å pore size) through auto‐sampler and then eluted into a C18 analytical column (75 µm x 250 mm, 2 µm particle size, 100 Å pore size). Mobile phase A (0.1% formic acid) and mobile phase B (90% ACN, 0.1% formic acid) were used to establish a 60 min seperation gradient. A constant flow rate was set at 300 nL min^−1^. Data was aquired using a spray votage of 2 kV, Ion funnel RF of 40, and ion transfer tube temperature of 320 °C. For DDA mode analysis, each scan cycle consisted of one full‐scan mass spectrum (Res. 60K, scan range 350–1500 m/z, AGC 300%, IT 20 ms) followed by MS/MS events (Res. 15K, AGC 100%, IT auto). Cycle time was set to 2 s. Isolation window was set at 1.6 Da. Dynamic exclution time was set to 35 s. Normalized collision energy was set at 30%. For Parallel Reaction Monitoring, each sample was analyzed under PRM with an isolation window of 1.6 Da. In all experiments, a full mass spectrum (Res. 60K, AGC target 300%, scan range 350 – 1500 m/z,IT 30 ms) was followed by up to 24 PRM scans (Res. 30K, AGC target 300%, IT 100 ms), as triggered by a unscheduled inclusion list. PRM data were manually curated within Skyline (version 21.1.0.278).

### Statistical Analysis

Differences between experimental and control groups were determined by Student's t test (where two groups of data were compared) or by two‐way ANOVA analysis (where more than two groups of data were compared).*p* values < 0.05 were considered statistically significant. For animal survival analysis, the Kaplan Meier method was adopted to generate graphs, and the survival curves were analysed with log‐rank analysis.

## Conflict of Interest

The authors declare no conflict of interest.

## Author Contributions

B.Z. and D.L. designed and supervised the study; Z.‐D.Z., H.‐X.L., and S.‐Q.Y. performed the experiments; H.G. and T.L. performed mass spectrometry assays; Z.T. and Y.‐Y.G. helped with the mouse experiments; B.Z., D.L., and Z.‐D.Z. wrote the paper. All authors analyzed the data.

## Supporting information

Supporting Information

Supplemental Table 1

Supplemental Table 2

## Data Availability

The data that support the findings of this study are available from the corresponding author upon reasonable request.
