# Supplementary material for: RNF115 Inhibits the Post‐ER Trafficking of TLRs and TLRs‐Mediated Immune Responses by Catalyzing K11‐Linked Ubiquitination of RAB1A and RAB13
Source: Adv Sci (Weinh). 2022 Mar 28;9(16):2105391. doi: 10.1002/advs.202105391 (PMC9165487; doi:10.1002/advs.202105391)
Supplement: Supplementary file 1 — Supporting Information [file ADVS-9-2105391-s001.pdf]

ADVANCED SCIENCE
Open Access

Supporting Information

for *Adv. Sci.*, DOI 10.1002/advs.202105391

RNF115 Inhibits the Post-ER Trafficking of TLRs and TLRs-Mediated Immune Responses by Catalyzing K11-Linked Ubiquitination of RAB1A and RAB13

Zhi-Dong Zhang, Hong-Xu Li, Hu Gan, Zhen Tang, Yu-Yao Guo, Shu-Qi Yao, Tianzi Liuyu, Bo Zhong and Dandan Lin**

Supporting Information

RNF115 inhibits the post-ER trafficking of TLRs and TLR-mediated immune responses by catalyzing K11-linked ubiquitination of RAB1A and RAB13

Zhang et al

Figure S1

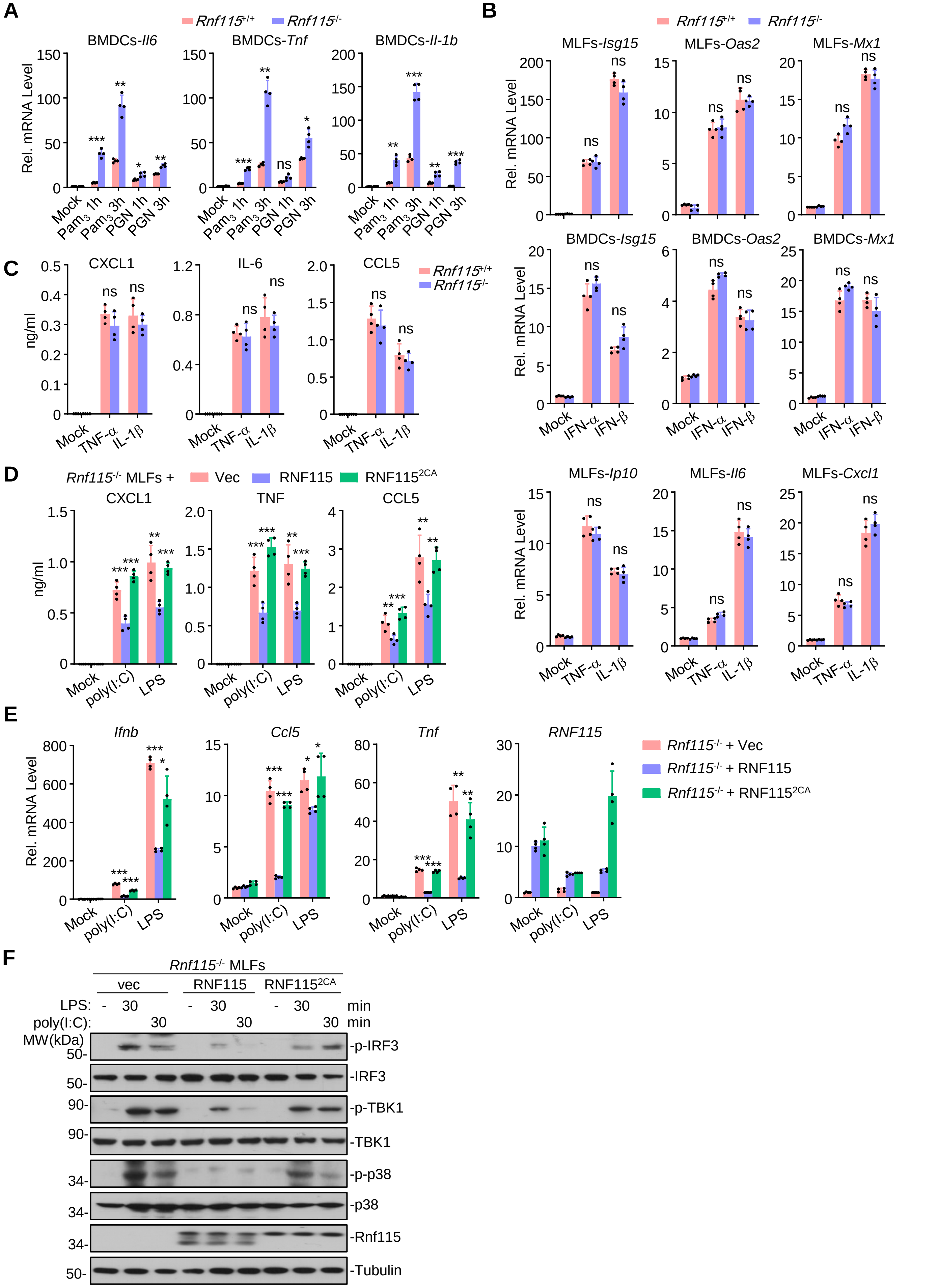

A
Rnf115+/+
Rnf115-/-
BMDCs-Il6
BMDCs-Tnf
BMDCs-Il-1b
Rel. mRNA Level
Mock
Pam3 1h
Pam3 3h
PGN 1h
PGN 3h
B
MLFs-Isg15
MLFs-Oas2
MLFs-Mx1
BMDCs-Isg15
BMDCs-Oas2
BMDCs-Mx1
IFN-α
IFN-β
C
CXCL1
IL-6
CCL5
ng/ml
TNF-α
IL-1β
D
Rnf115-/- MLFs +
Vec
RNF115
RNF115 2CA
CXCL1
TNF
CCL5
poly(I:C)
LPS
MLFs-Ip10
MLFs-Il6
MLFs-Cxcl1
E
Ifnb
Ccl5
Tnf
RNF115
Rnf115-/- + Vec
Rnf115-/- + RNF115
Rnf115-/- + RNF115 2CA
F
Rnf115-/- MLFs
vec
RNF115
RNF115 2CA
LPS:
poly(I:C):
min
MW(kDa)
-p-IRF3
-IRF3
-p-TBK1
-TBK1
-p-p38
-p38
-Rnf115
-Tubulin

Figure S1 RNF115 negatively regulates TLR-induced signaling in a manner dependent on its enzymatic activity.
(A) qPCR analysis of *Ifnb*, *Isg15*, or *Ccl5* mRNA in *Rnf115*$^{+/+}$ and *Rnf115*$^{-/-}$ BMDCs stimulated with Pam_3CSK_4 (1 μg/ml) or PGN (1 μg/ml) for 0, 1, or 3 h.
(B) qPCR analysis of *Oas2*, *Isg15*, or *Mx1* mRNA and *Ip10*, *Il6*, or *Cxcl1* in *Rnf115*$^{+/+}$ and *Rnf115*$^{-/-}$ MLFs or BMDCs stimulated with IFN-α (10 ng/ml) or IFN-β (10 ng/ml) (upper and middle graphs) and TNF-α (10 ng/ml) or IL-1β (10 ng/ml) (lower graphs) for 0 or 4 h, respectively.
(C) ELISA analysis of CXCL1 and IL-6 in the supernantants of *Rnf115*$^{+/+}$ and *Rnf115*$^{-/-}$ BMDCs stimulated with TNF-α (10 ng/ml) or IL-1β (10 ng/ml) for 0 or 6 h.
(D, E) ELISA analysis of CXCL1, TNF, and CCL5 (D) or qPCR analysis of *Ifnb*, *Ccl5*, *Tnf* or *Rnf115* mRNA (E) in *Rnf115*$^{-/-}$ MLFs reconstituted with the empty vector (Vec), RNF115, or RNF115(C228/C231A) (RNF115^{2CA}) followed by stimulation with LPS (1 μg/ml) or poly(I:C) (50 μg/ml) for 0 or 6 h.
(F) Immunoblot analysis of total and phosphorylated (p-)p38, IRF3, and TBK1, total RNF115, RNF115^{2CA}, and Tubulin in cells obtained in (D) stimulated with LPS (1 μg/ml) or poly(I:C) (50 μg/ml) for 0-30 min.
*$P < 0.05$, **$P < 0.01$, ***$P < 0.001$ and ns (not significant) (two-tailed Student's *t*-test). Data are representative of three independent experiments (graphs show mean ± SD in A-E).

Figure S2

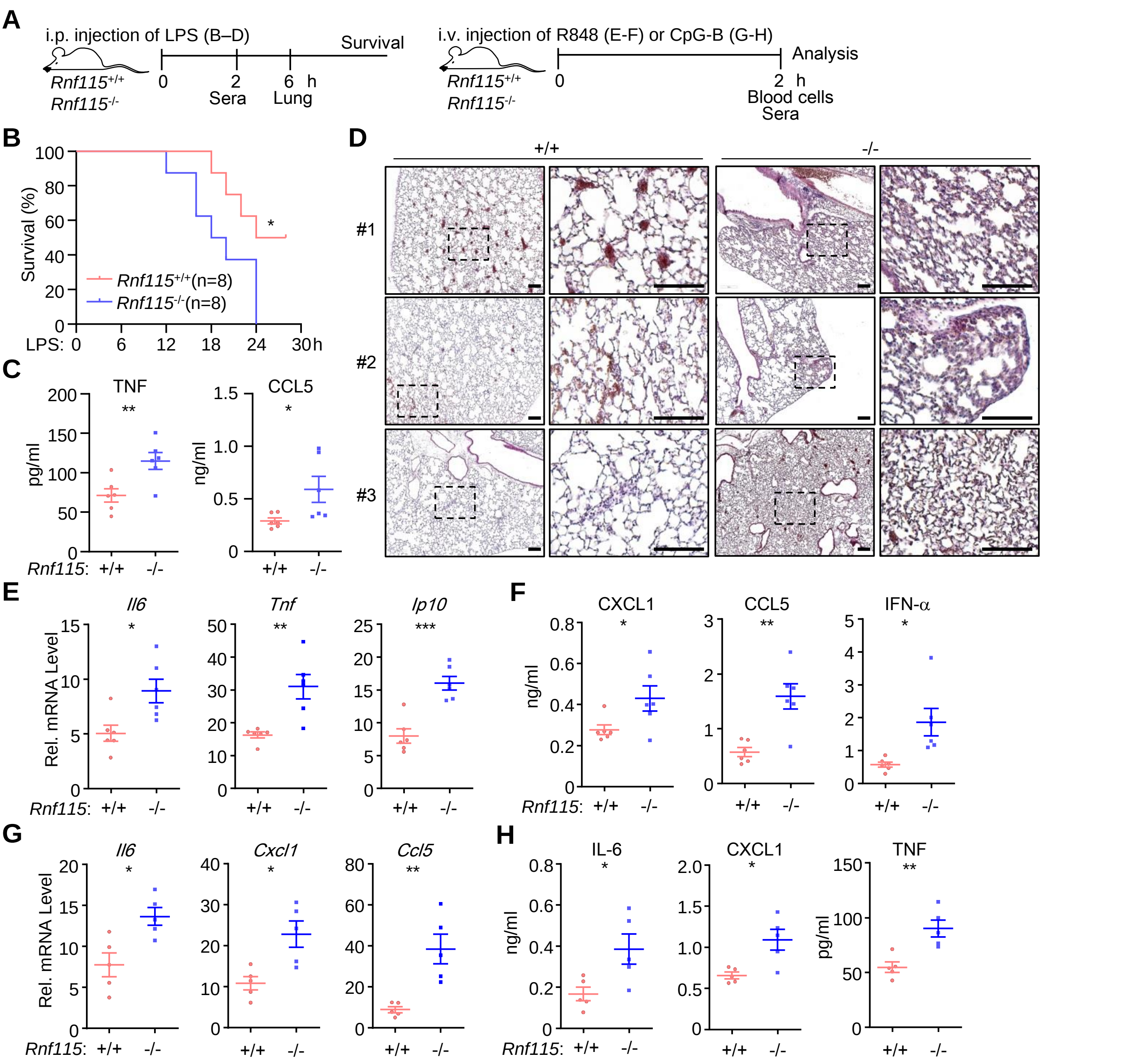

Figure S2 Knockout of RNF115 potentiated TLR-induced production of proinflammatory cytokines in mice.

(A) A scheme of LPS, R848, or CpG-B injection and analysis of *Rnf115*$^{+/+}$ and *Rnf115*$^{-/-}$ mice.

(B) Survival (Kaplan-Meier curve) of *Rnf115*$^{+/+}$ and *Rnf115*$^{-/-}$ mice (n=8) intraperitoneally injected with LPS (10 mg/g body weight) and monitored for 30 h.

(C, D) ELISA analysis of the sera (C) or hematoxylin-eosin staining (HE) of the lungs (D) from *Rnf115*$^{+/+}$ and *Rnf115*$^{-/-}$ mice (n=6) intraperitoneally injected with LPS (10 mg/g body weight) for 2 or 6 h, respectively.

(E, F) qPCR analysis of blood cells (E) or ELISA analysis of the sera (f) from *Rnf115*$^{+/+}$ and *Rnf115*$^{-/-}$ mice (n=6) intravenously injected with R848 (1 mg/g body weight) for 2 h.

(G, H) qPCR analysis of blood cells (G) or ELISA analysis of sera (H) from *Rnf115*$^{+/+}$ and *Rnf115*$^{-/-}$ mice (n=5) intravenously injected with CpG-B (1 mg/g body weight) for 2 h.

*$P < 0.05$ and **$P < 0.01$ (two-tailed Student's *t*-test). Scale bars represent 200 μm. Data are representative of two independent experiments (graphs show mean ± SD in B, C, E-H).

Figure S3

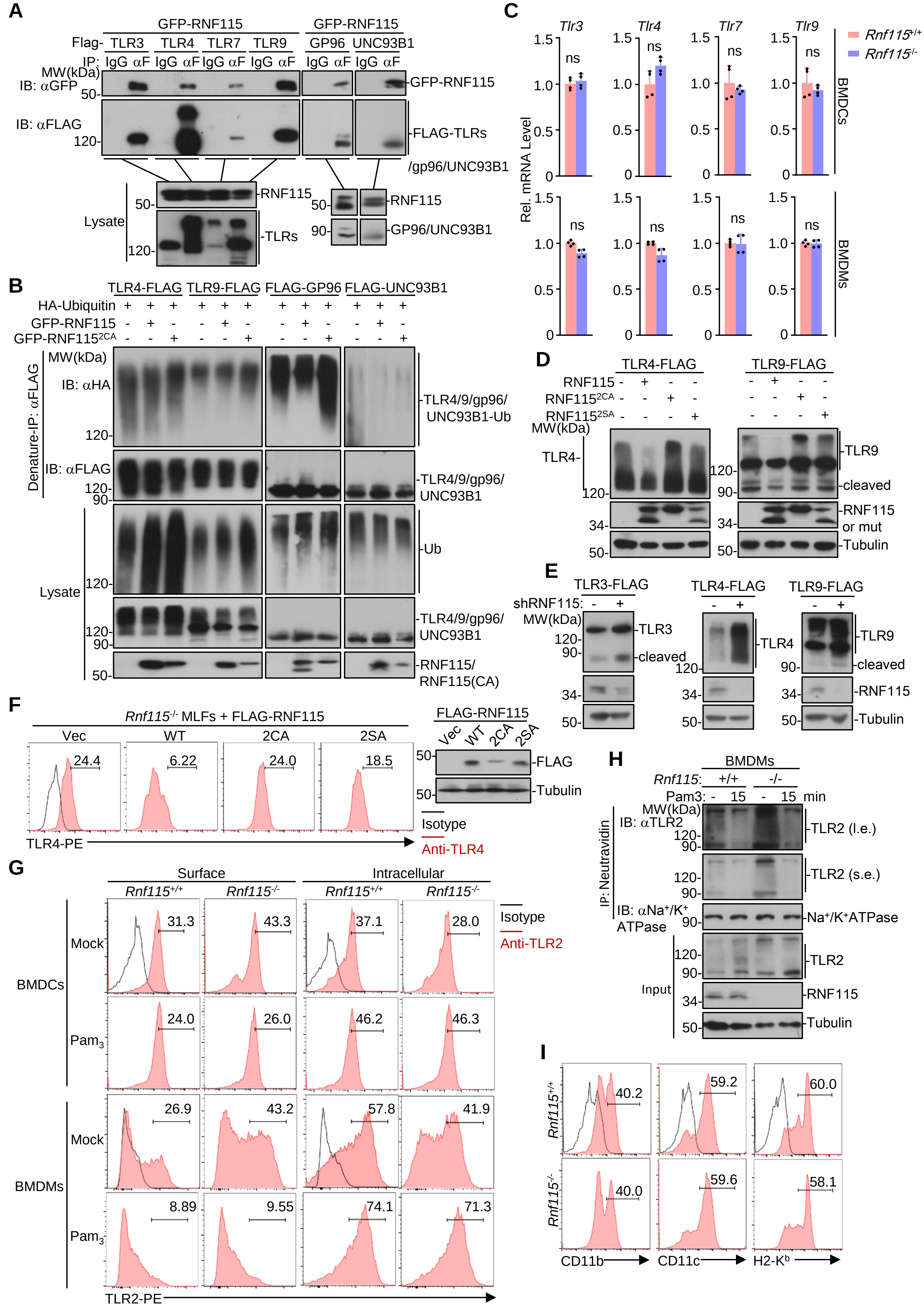
A
GFP-RNF115
Flag- TLR3 TLR4 TLR7 TLR9 GP96 UNC93B1
IP: IgG αF IgG αF IgG αF IgG αF IgG αF IgG αF
MW(kDa)
IB: αGFP
GFP-RNF115
IB: αFLAG
FLAG-TLRs /gp96/UNC93B1
Lysate
RNF115
TLRs
GP96/UNC93B1
B
TLR4-FLAG TLR9-FLAG FLAG-GP96 FLAG-UNC93B1
HA-Ubiquitin
GFP-RNF115
GFP-RNF115^2CA
Denature-IP: αFLAG
IB: αHA
IB: αFLAG
TLR4/9/gp96/ UNC93B1-Ub
TLR4/9/gp96/ UNC93B1
Ub
RNF115/ RNF115(CA)
C
Tlr3 Tlr4 Tlr7 Tlr9
Rel. mRNA Level
ns
BMDCs
BMDMs
Rnf115^+/+
Rnf115^-/-
D
TLR4-FLAG TLR9-FLAG
RNF115
RNF115^2CA
RNF115^2SA
TLR4-
TLR9
cleaved
RNF115 or mut
Tubulin
E
TLR3-FLAG TLR4-FLAG TLR9-FLAG
shRNF115:
TLR3
TLR4
TLR9
cleaved
RNF115
Tubulin
F
Rnf115^-/- MLFs + FLAG-RNF115
Vec WT 2CA 2SA
24.4 6.22 24.0 18.5
TLR4-PE
FLAG-RNF115
FLAG
Tubulin
Isotype
Anti-TLR4
G
Surface Intracellular
Rnf115^+/+ Rnf115^-/- Rnf115^+/+ Rnf115^-/-
BMDCs
Mock 31.3 43.3 37.1 28.0
Pam3 24.0 26.0 46.2 46.3
BMDMs
Mock 26.9 43.2 57.8 41.9
Pam3 8.89 9.55 74.1 71.3
TLR2-PE
Isotype
Anti-TLR2
H
BMDMs
Rnf115: +/+ -/-
Pam3: - 15 - 15 min
IP: Neutravidin
IB: αTLR2
TLR2 (l.e.)
TLR2 (s.e.)
IB: αNa+/K+ ATPase
Na+/K+ATPase
Input
TLR2
RNF115
Tubulin
I
Rnf115^+/+
Rnf115^-/-
40.2 59.2 60.0
40.0 59.6 58.1
CD11b CD11c H2-K^b

Figure S3 RNF115 inhibits the glycosylation the post-ER trafficking of TLRs dependently on its enzymatic activity.

(A) Immunoprecipitation (with anti-FLAG) and immunoblot analysis (with anti-GFP or anti-FLAG) of HEK293 cells transfected with the indicated plasmids for 24 h.

(B) Denature-immunoprecipitation (Denature-IP) (with anti-FLAG) and immunoblot analysis (with anti-FLAG, anti-HA, or anti-GFP) of HEK293 cells transfected with the indicated plasmids for 24 h.

(C) qPCR analysis of the *Tlr3*, *Tlr4*, *Tlr7*, or *Tlr9* mRNA in *Rnf115*$^{+/+}$ and *Rnf115*$^{-/-}$ BMDCs or BMDMs.

(D) Immunoblot analysis of HEK293 cells that were transfected with plasmids encoding empty vector, RNF115, RNF115^{2CA}, or RNF115(S132/133A) (RNF115^{2SA}) together with TLR4-FLAG (left panels) or TLR9-FLAG (right panels) for 24 h, respectively.

(E) Immunoblot analysis of HEK293 cells that were transfected with a control shRNA or shRNAF115 together with TLR3-FLAG (left panels), TLR4-FLAG (middle panels), or TLR9-FLAG (right panels) for 36 h, respectively.

(F) Flow cytometry analysis of *Rnf115*$^{-/-}$ MLFs reconstituted with the empty vector (Vec), RNF115, RNF115^{2CA}, or RNF115^{2SA} followed by staining with anti-TLR4-PE (red line) or an isotype control (black line). The expression levels of RNF115, RNF115^{2CA}, or RNF115^{2SA} were determined by immunoblot analysis.

(G) Flow cytometry analysis of the cell surface and intracellular TLR2 of *Rnf115*$^{+/+}$ and *Rnf115*$^{-/-}$ BMDCs or BMDMs that were left unstimulated or stimulated with Pam_3CSK_4 (1 μg/ml) for 15 min.

(H) *Rnf115*$^{+/+}$ and *Rnf115*$^{-/-}$ BMDMs were left unstimulated or stimulated with Pam_3CSK_4 (1 μg/ml) for 15 min followed by incubation with biotin for 30 min. The non-binding biotin was quenched by glycine for 20 min and the cell lysate was prepared followed by immunoprecipitation (with Neutraavidin agarose) and immunoblot analysis (with anti-TLR4, anti-Na^+/K^+ ATPase, anti-RNF115, or anti-Tubulin). l.e., long-time exposure. s.e., short-time exposure.

(I) Flow cytometry analysis of *Rnf115*$^{+/+}$ and *Rnf115*$^{-/-}$BMDCs that were stained with H2-K^b (MHC-I), CD11b, or CD11c or an isotype control.

ns (not significant) (two-tailed Student's *t*-test). Data are representative of three independent experiments (graphs show mean ± SD in C).

Figure S4

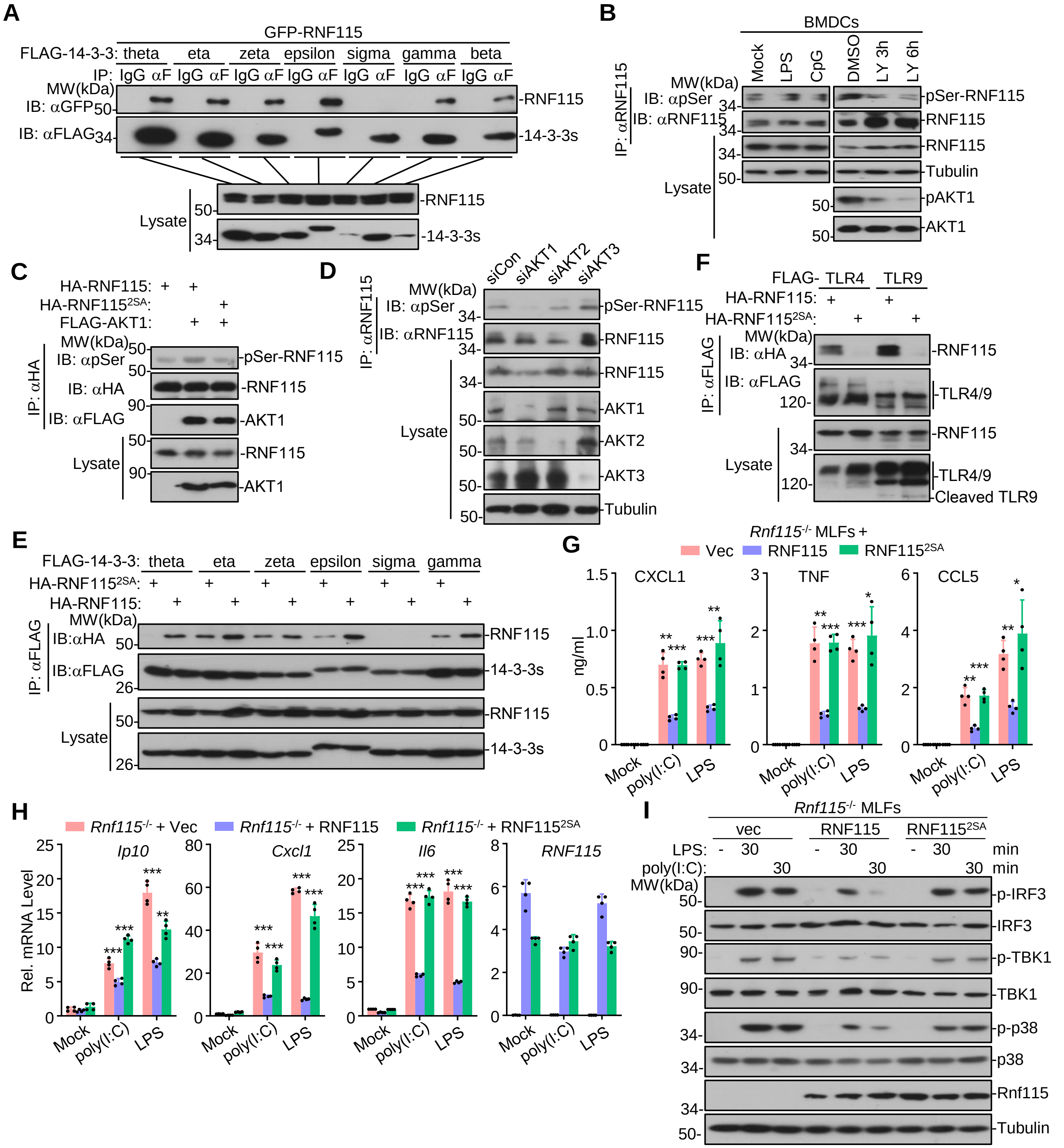

Figure S4 The phosphorylation on Ser132/133 of RNF115 is required for restricting TLR signaling.

(A) Immunoprecipitation (with IgG or anti-FLAG) and immunoblot analysis (with anti-GFP or anti-FLAG) of HEK293 cells transfected with indicated plasmids for 24 h.

(B) Immunoprecipitation (with anti-RNF115) and immunoblot analysis (with anti-RNF115, anti-pSer, anti-pAKT1, anti-AKT1, or anti-Tubulin) of BMDCs left unstimulated or stimulated with LPS (1 μg/ml) or CpG-B (5 μM) for 15 min (left panels) or treated with LY294002 for 0-6 h (right panels).

(C) Immunoprecipitation (with anti-HA) and immunoblot analysis (with anti-HA, anti-pSer, or anti-AKT1) of HEK293 cells transfected with plasmids encoding HA-RNF115 or HA-RNF115^{2SA} and FLAG-AKT1 for 24 h.

(D) Immunoprecipitation (with anti-RNF115) and immunoblot analysis (with anti-RNF115, anti-pSer, anti-AKT1, anti-AKT2, anti-AKT3, or anti-Tubulin) of MLF cells transfected with siCon, siAKT1, siAKT2, or siAKT3 for 48 h.

(E) Immunoprecipitation (with anti-FLAG) and immunoblot analysis (with anti-HA or anti-FLAG) of HEK293 cells transfected with plasmids encoding HA-RNF115 or HA-RNF115^{2SA}) together with FLAG-14-3-3 chaperones (theta, eta, zeta, epsilon, sigma, or gamma) for 24 h.

(F) Immunoprecipitation (with anti-FLAG) and immunoblot analysis (with anti-HA or anti-FLAG) of HEK293 cells transfected with plasmids encoding HA-RNF115 or HA-RNF115^{2SA} and TLR4-FLAG or TLR9-FLAG for 24 h.

(G) qPCR analysis of *Ip10*, *Cxcl1*, *Il6*, or *Rnf115* mRNA in *Rnf115*$^{-/-}$ MLFs reconstituted with the empty vector (Vec), RNF115, or RNF115^{2SA} followed by stimulation with LPS (1 μg/ml) or poly(I:C) (50 μg/ml) for 0-3 h.

(H) ELISA analysis of CXCL1, TNF, and CCL5 in cells obtained in (f) stimulated with LPS or poly(I:C) for 0-6 h.

(I) Immunoblot analysis of total and phosphorylated (p-) p38, IRF3 and TBK1, and RNF115, RNF115^{2SA}, and β-Tubulin in cells obtained in (F) left unstimulated or stimulated with LPS (1 μg/ml) or poly(I:C) (50 μg/ml) for 30min.

$^{*}P < 0.05$, $^{**}P < 0.01$, and $^{***}P < 0.001$ (two-tailed Student's *t*-test). Data are representative of three independent experiments (graphs show mean ± SD in G-H).

Figure S5

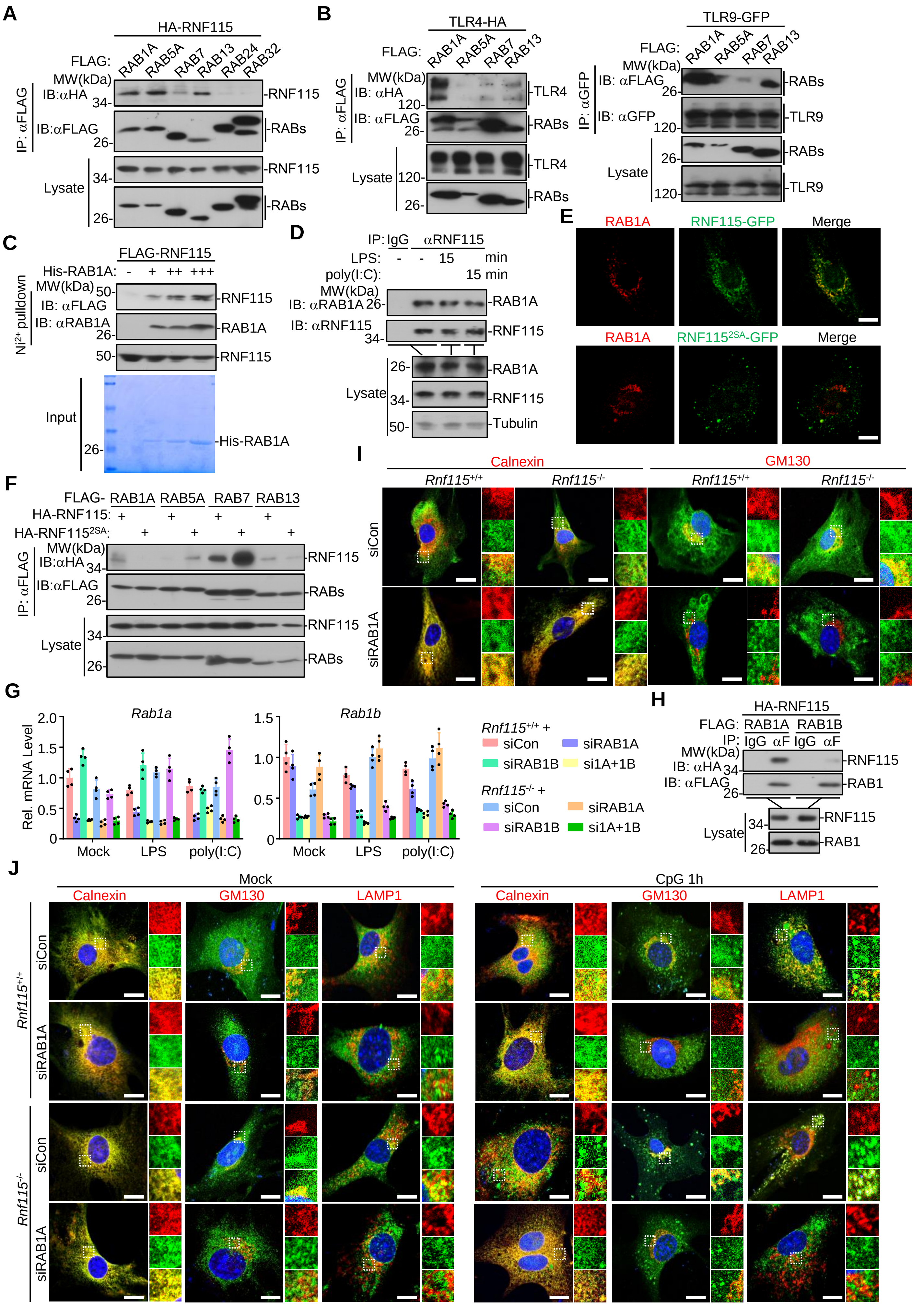

A
HA-RNF115
FLAG: RAB1A RAB5A RAB7 RAB13 RAB24 RAB32
MW(kDa)
IP: αFLAG
IB:αHA
IB:αFLAG
Lysate
RNF115
RABs
B
TLR4-HA
FLAG: RAB1A RAB5A RAB7 RAB13
IB: αHA
IB: αFLAG
TLR4
TLR9-GFP
IP: αGFP
IB: αGFP
TLR9
C
FLAG-RNF115
His-RAB1A: - + ++ +++
Ni2+ pulldown
IB: αRAB1A
RAB1A
Input
His-RAB1A
D
IP: IgG αRNF115
LPS: - - 15 min
poly(I:C): 15 min
IB: αRNF115
Tubulin
E
RAB1A
RNF115-GFP
Merge
RNF115 2SA-GFP
F
FLAG- RAB1A RAB5A RAB7 RAB13
HA-RNF115: + + + +
HA-RNF115 2SA: + + + +
G
Rel. mRNA Level
Rab1a
Rab1b
Mock
LPS
poly(I:C)
Rnf115+/+ +
siCon
siRAB1A
siRAB1B
si1A+1B
Rnf115-/- +
H
HA-RNF115
FLAG: RAB1A RAB1B
IP: IgG αF IgG αF
RAB1
I
Calnexin
GM130
Rnf115+/+
Rnf115-/-
siCon
siRAB1A
J
Mock
CpG 1h
LAMP1

Figure S5 RNF115 interacts with RAB1A.

(A) Immunoprecipitation (with anti-FLAG) and immunoblot analysis (with anti-FLAG or anti-HA) of HEK293 cells transfected with plasmids encoding HA-RNF115 and FLAG-RABs (RAB1A, RAB5A, RAB7A, RAB13, RAB24, or RAB32) for 24 h.

(B) Immunoprecipitation with anti-FLAG (left panels) or anti-GFP (right panels) and immunoblot analysis (with anti-FLAG, anti-HA, or anti-GFP) of HEK293 cells transfected with plasmids encoding HA-TLR4 or GFP-TLR9 and FLAG-RABs (RAB1A, RAB5A, RAB7A, or RAB13) for 24 h.

(C) Ni^{2+} agarose pulldown assay of His-RAB1A (0, 0.5 μg, 1 μg, 2 μg, respectively) and FLAG-RNF115 eluted from anti-FLAG precipitates by FLAG peptide of HEK293 cells transfected with FLAG-RNF115.

(D) Immunoprecipitation (with IgG or anti-RNF115) and immunoblot analysis (with anti-RAB1A, RNF115, or Tubulin) of BMDCs unstimulated or stimulated with LPS (1 μg/ml) or poly(I:C) (50 μg/ml) for 15 min.

(E) Immunofluorescent staining RAB1A (red) and confocal microscopy imaging of *Rnf115*$^{-/-}$ MLFs reconstituted with GFP-RNF115 or GFP-RNF115^{2SA}.

(F) Immunoprecipitation (with anti-FLAG) and immunoblot analysis (with anti-FLAG or anti-HA) of HEK293 cells transfected with plasmids encoding HA-RNF115 or HA-RNF115^{2SA} and FLAG-RABs (RAB1A, RAB5A, RAB7A, or RAB13) for 24 h.

(G) qPCR analysis of *Rab1a* or *Rab1b* mRNA in *Rnf115*$^{+/+}$ and *Rnf115*$^{-/-}$ MLFs transfected with siCon, siRAB1A, or siRAB1B for 48 h followed by treatment with LPS (1 μg/ml) or poly(I:C) (50 μg/ml) for 0-3 h.

(H) Immunoprecipitation (with anti-FLAG) and immunoblot analysis (with anti-FLAG or anti-HA) of HEK293 cells transfected with plasmids encoding HA-RNF115 and FLAG-RAB1A or RAB1B for 24 h.

(I) Immunofluorescent staining Calnexin (red), GM130 (red), or TLR4 (green) and confocal microscopy analysis in *Rnf115*$^{+/+}$ and *Rnf115*$^{-/-}$ MLFs transfected with siCon or siRAB1A for 48 h.

(J) The *Rnf115*$^{+/+}$ or *Rnf115*$^{-/-}$ MLFs were reconstituted with TLR9-GFP followed by transfection with siCon or siRAB1A for 48 h. Cells were left unstimulated or stimulated by CpG-B (5 μM) for 1 h followed by staining with anti-Calnexin (red), anti-GM130 (red), or anti-LAMP1 (red) and confocal microscopy analysis. Scale bars represent 5 μm. Data are representative of three independent experiments.

Figure S6

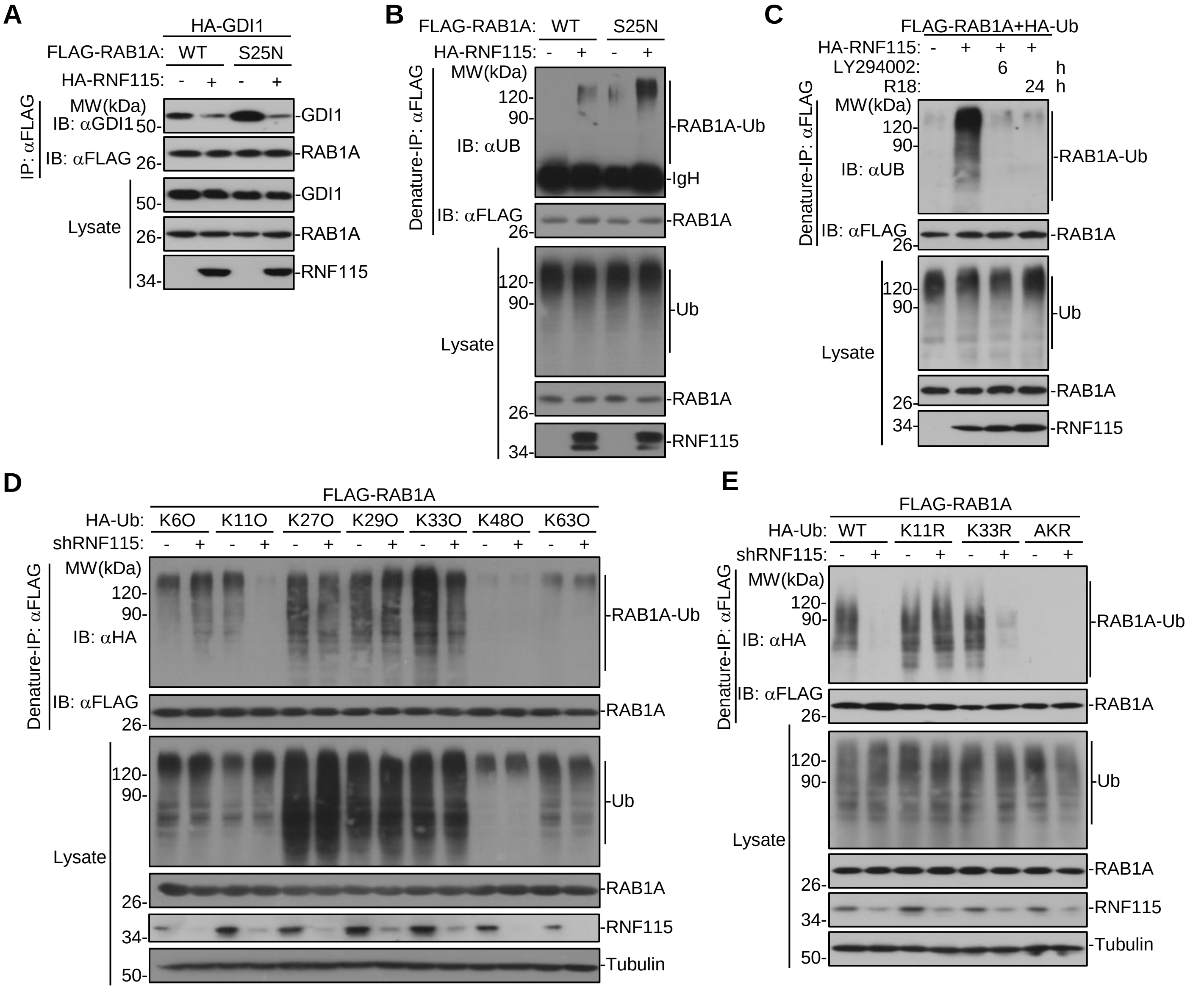

Figure S6 RNF115 catalyzes K11-linked ubiquitination of RAB1A.

(A) Immunoprecipitation (with anti-FLAG) and immunoblot analysis (with anti-FLAG, anti-HA, or anti-GDI1) of HEK293 cells transfected with the plasmids encoding HA-GDI1 and FLAG-RAB1A or FLAG-RAB1A(S25N) together with an empty vector or HA-RNF115 for 24 h.

(B) Denature-IP (with anti-FLAG) and immunoblot analysis (with anti-FLAG, anti-HA, or anti-Ub) of HEK293 cells transfected with plasmids encoding an empty vector or HA-RNF115 together with FLAG-RAB1A or FLAG-RAB1A(S25N) for 24 h.

(C) Denature-IP (with anti-FLAG) and immunoblot analysis (with anti-FLAG or anti-HA) of HEK293 cells transfected with plasmids encoding HA-Ubiquitin, and an empty vector, HA-RNF115, or FLAG-RAB1A for 18 h, followed by treatment with LY294002 or R18 for 6 or 24 h, respectively.

(D) Denature-IP (with anti-FLAG) and immunoblot analysis (with anti-FLAG, anti-HA, anti-RNF115, or anti-Tubulin) of HEK293 cells transfected with plasmids encoding FLAG-RAB1A and a control shRNA or shRNF115 together with HA-ubiquitin, or ubiquitin mutants for 36 h.

(E) Denature-IP (with anti-FLAG) and immunoblot analysis (with anti-FLAG, anti-HA, anti-RNF115, or anti-Tubulin) of HEK293 cells transfected with plasmids encoding FLAG-RAB1A and a control shRNA or shRNF115 together with HA-ubiquitin, or ubiquitin mutants for 36 h.

Data are representative of three independent experiments.

Figure S7

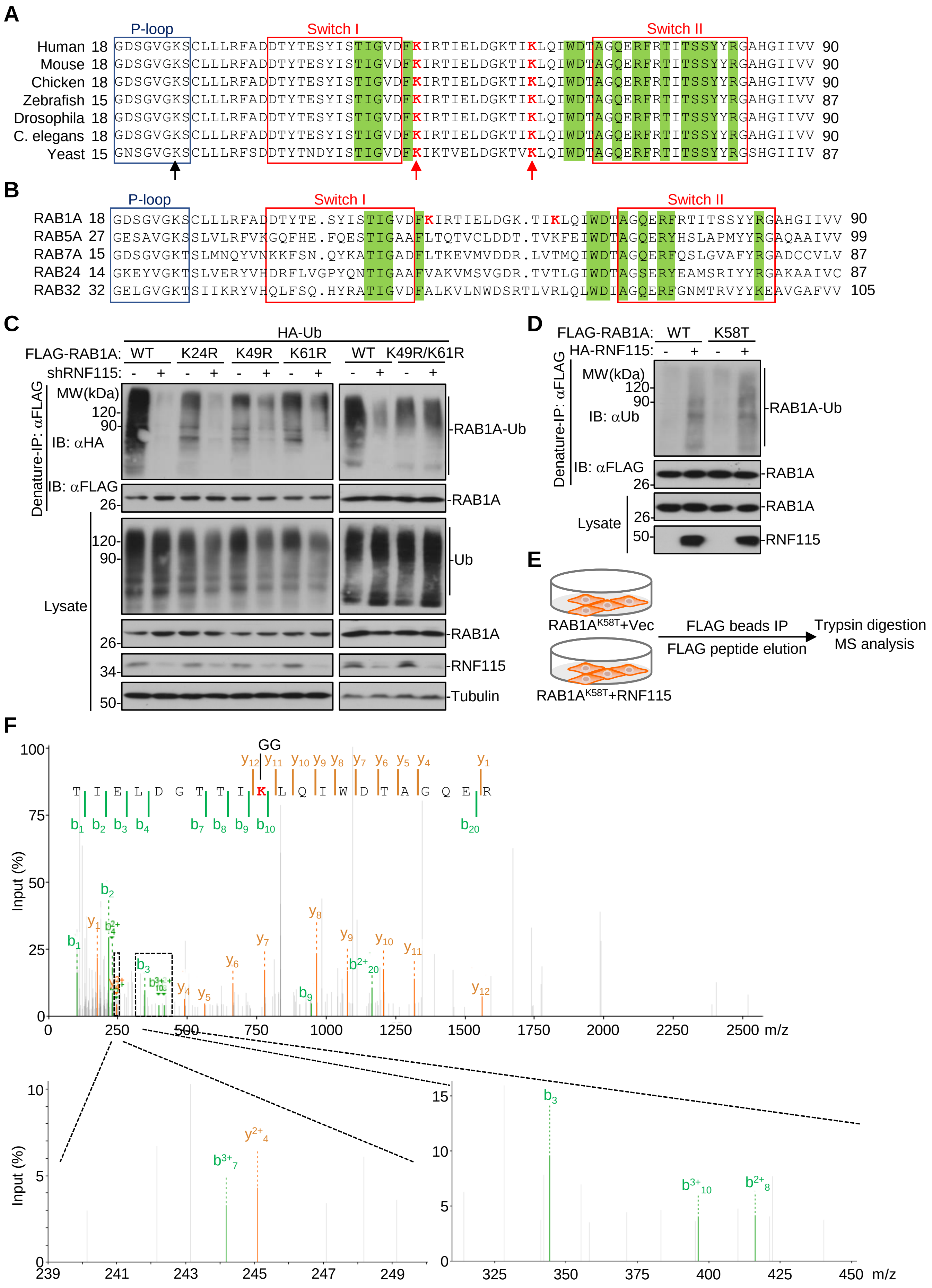

A
P-loop
Switch I
Switch II
Human 18 GDSGVGKSCLLLRFADDTYTESYISTIGVDFKIRTIELDGKTIKLQIWDTAGQERFRTITSSYYRGAHGIIVV 90
Mouse 18 GDSGVGKSCLLLRFADDTYTESYISTIGVDFKIRTIELDGKTIKLQIWDTAGQERFRTITSSYYRGAHGIIVV 90
Chicken 18 GDSGVGKSCLLLRFADDTYTESYISTIGVDFKIRTIELDGKTIKLQIWDTAGQERFRTITSSYYRGAHGIIVV 90
Zebrafish 15 GDSGVGKSCLLLRFADDTYTESYISTIGVDFKIRTIELDGKTIKLQIWDTAGQERFRTITSSYYRGAHGIIVV 87
Drosophila 18 GDSGVGKSCLLLRFADDTYTESYISTIGVDFKIRTIELDGKTIKLQIWDTAGQERFRTITSSYYRGAHGIIVV 90
C. elegans 18 GDSGVGKSCLLLRFADDTYTESYISTIGVDFKIRTIELDGKTIKLQIWDTAGQERFRTITSSYYRGAHGIIVV 90
Yeast 15 GNSGVGKSCLLLRFSDDTYTNDYISTIGVDFKIKTVELDGKTVKLQIWDTAGQERFRTITSSYYRGSHGIIIV 87
B
P-loop
Switch I
Switch II
RAB1A 18 GDSGVGKSCLLLRFADDTYTE.SYISTIGVDFKIRTIELDGK.TIKLQIWDTAGQERFRTITSSYYRGAHGIIVV 90
RAB5A 27 GESAVGKSSLVLRFVKGQFHE.FQESTIGAAFLTQTVCLDDT.TVKFEIWDTAGQERYHSLAPMYYRGAQAAIVV 99
RAB7A 15 GDSGVGKTSLMNQYVNKKFSN.QYKATIGADFLTKEVMVDDR.LVTMQIWDTAGQERFQSLGVAFYRGADCCVLV 87
RAB24 14 GKEYVGKTSLVERYVHDRFLVGPYQNTIGAAFVAKVMSVGDR.TVTLGIWDTAGSERYEAMSRIYYRGAKAAIVC 87
RAB32 32 GELGVGKTSIIKRYVHQLFSQ.HYRATIGVDFALKVLNWDSRTLVRLQLWDIAGQERFGNMTRVYYKEAVGAFVV 105
C
HA-Ub
FLAG-RAB1A: WT K24R K49R K61R WT K49R/K61R
shRNF115: - + - + - + - + - + - +
MW(kDa)
120-
90-
IB: αHA
RAB1A-Ub
IB: αFLAG
26-
RAB1A
Denature-IP: αFLAG
120-
90-
Ub
Lysate
26-
RAB1A
34-
RNF115
50-
Tubulin
D
FLAG-RAB1A: WT K58T
HA-RNF115: - + - +
MW(kDa)
120-
90-
IB: αUb
RAB1A-Ub
IB: αFLAG
26-
RAB1A
Denature-IP: αFLAG
Lysate
26-
RAB1A
50-
RNF115
E
RAB1A^K58T+Vec
RAB1A^K58T+RNF115
FLAG beads IP
FLAG peptide elution
Trypsin digestion
MS analysis
F
GG
T I E L D G T T I K L Q I W D T A G Q E R
y12 y11 y10 y9 y8 y7 y6 y5 y4 y1
b1 b2 b3 b4 b7 b8 b9 b10 b20
Input (%)
100
75
50
25
0
0 250 500 750 1000 1250 1500 1750 2000 2250 2500 m/z
b2 y1 b2+4 b1 y8 y7 y9 y10 y11 b2+20 y6 b3 b3+10 y4 y5 b9 y12
10
5
0
239 241 243 245 247 249
b3+7 y2+4
15
10
5
0
325 350 375 400 425 450 m/z
b3 b3+10 b2+8

Figure S7 RNF115 catalyzes ubiquitination on Lys49 and Lys61 of RAB1A.

(A) An alignment of RAB1A from different species. The blue rectangle indicates the P-loop and red rectangles indicate the switch I and II domains. The black arrow indicates the conserved Lys24 and the red arrows indicate the ubiquitinated Lys49 and Lys61. The green shadows indicate the interface associating with GDI.

(B) An alignment of representative RAB proteins. The blue rectangle indicates the P-loop and red rectangles indicate the switch I and II domains. The green shadows indicate conserved residues in the switch I and II domains.

(C) Denature-IP (with anti-FLAG) and immunoblot analysis (with anti-FLAG, anti-HA, anti-RNF115, or anti-Tubulin) of HEK293 cells transfected with plasmids encoding HA-ubiquitin and FLAG-tagged RAB1A or RAB1Amutants together with a control shRNA or shRNF115 for 36 h.

(D) Denature-IP (with anti-FLAG) and immunoblot analysis (with anti-FLAG, anti-HA, and anti-Ub) of HEK293 cells transfected with plasmids encoding FLAG-RAB1A, RAB1A^{K58T} together with a empty control vector or HA-RNF115 for 24 h.

(E) Strategy of analysis on RNF115-mediated ubiquitinated sites of RAB1A. RAB1A^{K58T} is purified from HEK293 cells transfected with FLAG-RAB1A^{K58T} together with a empty control vector or HA-RNF115 and followed by immunoprecipitation (with anti-FLAG agarose) and elution (with 3 $\times$ FLAG peptide), and is digested by trypsin and subsequently analysed by mass spectrometer.

(F) Mass spectrometry analysis of the RNF115-mediated ubiquitinated sites of RAB1A.

Data are representative of three independent experiments.

Figure S8

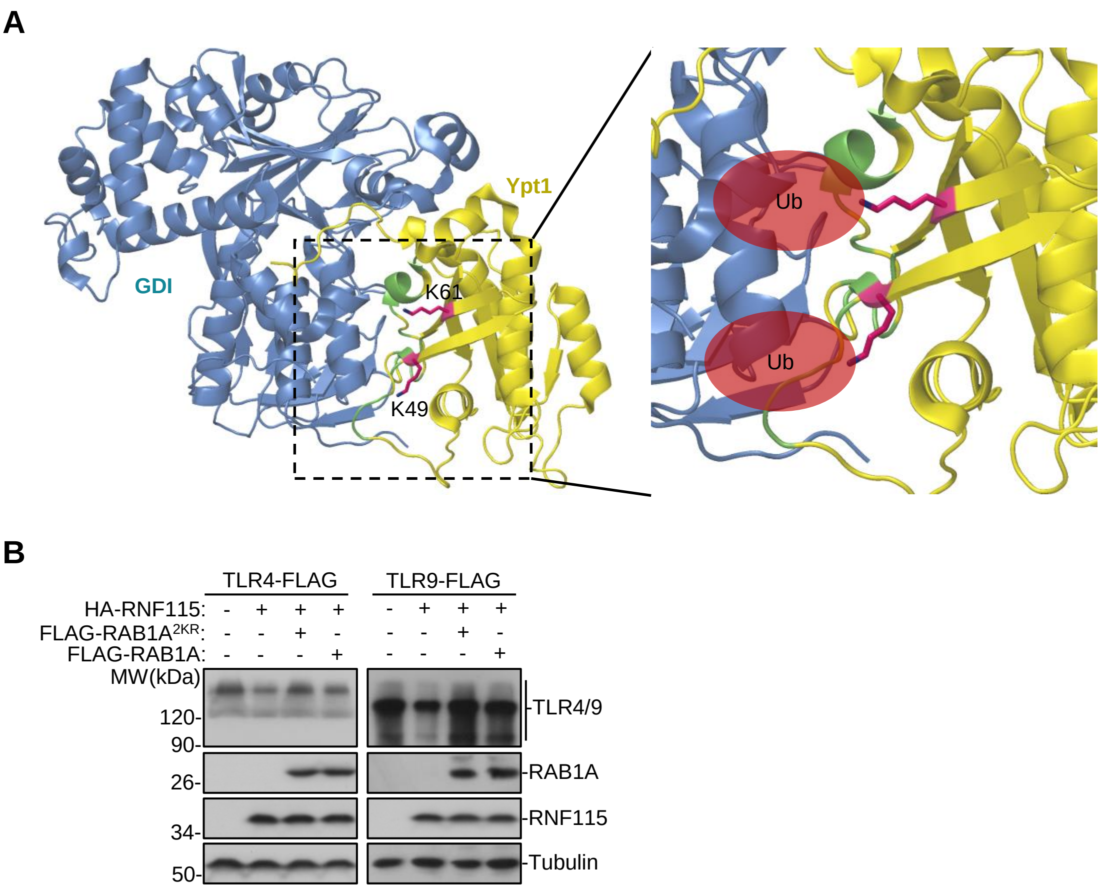

Figure S8 RAB1A$^{K49/61R}$ promotes TLRs trafficking and TLRs-mediated signaling.
(A) Structure of Ypt1 (yellow)-GDI (blue) complexes. Ubiquitin (red) modification on K49 (pink) and K61 (pink) of Ypt1 might interfere with two interfaces (green) interacting with GDI.
(B) Immunoblot analysis (with anti-FLAG, anti-HA, or anti-tubulin) in HEK293 cells transfected with plasmids encoding TLR4-FLAG (left panels), TLR9-FLAG (right panels), and HA-RNF115, FLAG-tagged RAB1A or RAB1Amutants for 24 h.
Data are representative of three independent experiments.

Figure S9

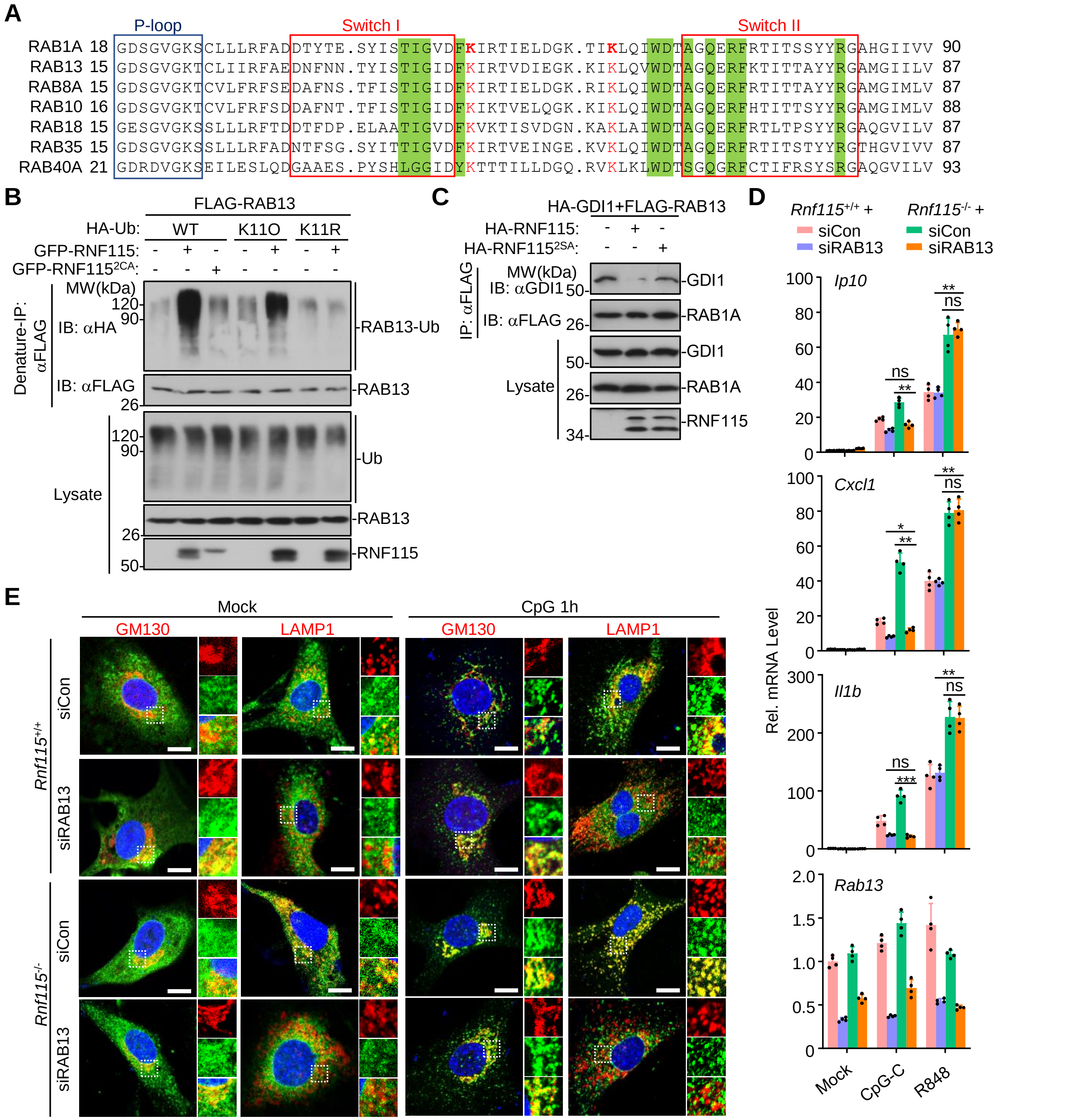

Figure S9 RNF115 catalyzes ubiquitination on Lys46 and Lys58 of RAB13.

(A) An alignment of different RABs. The blue rectangle indicates the P-loop and red rectangles indicate the switch I and II domains. The red arrows indicate the ubiquitinated Lys49 and Lys61. The green shadows indicate the interface associating with GDI.

(B) Denature-IP (with anti-FLAG) and immunoblot analysis (with anti-FLAG, anti-HA, or anti-GFP) of HEK293 cells transfected with plasmids encoding HA-ubi (WT, K11O, or K11R), FLAG-RAB13, GFP-RNF115, or RNF115^{2CA} for 24 h.

(C) Immunoprecipitation with anti-FLAG and immunoblot analysis (with anti-FLAG, anti-HA, or anti-GDI1) of HEK293 cells transfected with plasmids encoding HA-GDI1 and FLAG-RAB13, HA-RNF115, or HA-RNF115^{2SA} for 24 h.

(D) qPCR analysis of *Ip10*, *Il1b*, *Cxcl1*, or *Rab13* mRNA in *Rnf115*$^{+/+}$ and *Rnf115*$^{-/-}$ pDCs transfected with siCon, siRAB13 for 48 h followed by treatment with R848 (1 μg/ml) or CpG-B (5 μM) for 0-3 h.

(E) The *Rnf115*$^{+/+}$ or *Rnf115*$^{-/-}$ MLFs were reconstituted with TLR9-GFP followed by transfection with siCon or siRAB13 for 48 h. Cells were left unstimulated or stimulated by CpG-B (5 μM) for 1 h followed by staining with anti-GM130 (red) or anti-LAMP1 (red) and confocal microscopy analysis.

$^*P < 0.05$, $^{**}P < 0.01$, $^{***}P < 0.001$, and ns (not significant) (two-tailed Student's *t*-test). Scale bars represent 5 μm. Data are representative of three independent experiments (graphs show mean ± SD in D).
