# Supplementary material for: RNF115 Inhibits the Post‐ER Trafficking of TLRs and TLRs‐Mediated Immune Responses by Catalyzing K11‐Linked Ubiquitination of RAB1A and RAB13
Source: Adv Sci (Weinh). 2022 Mar 28;9(16):2105391. doi: 10.1002/advs.202105391 (PMC9165487; doi:10.1002/advs.202105391)
Supplement: Supplementary file 3 — Supplemental Table 2 [file ADVS-9-2105391-s003.pdf]

ADVANCED SCIENCE
Open Access

Supporting Information

for *Adv. Sci.*, DOI 10.1002/advs.202105391

RNF115 Inhibits the Post-ER Trafficking of TLRs and TLRs-Mediated Immune Responses by Catalyzing K11-Linked Ubiquitination of RAB1A and RAB13

Zhi-Dong Zhang, Hong-Xu Li, Hu Gan, Zhen Tang, Yu-Yao Guo, Shu-Qi Yao, Tianzi Liuyu, Bo Zhong and Dandan Lin**

Supplementary Table 2. qPCR primer sequences

Gene	Forward	Reverse
β-Actin	ACGGCCAGGTCATCACTATT	TGGCATAGAGGTCTTTACGGA
Ifna1	GGATGTGACCTTCCTCAGACTC	ACCTTCTCCTGCGGGAATCCAA
Ifnb	TCCTGCTGTGCTTCTCCACCA	AAGTCCGCCCTGTAGGTGAGG
Isg15	GGCCACAGCAACATCTATGA	ACTGGGGCTTTAGGCCATAC
Mx1	GACCATAGGGGTCTTGACCAA	AGACTTGCTCTTTCTGAAAAGCC
Oas2	TTGAAGAGGAATACATGCGGAAG	GGGTCTGCATTACTGGCACTT
Ccl5	CTGCTGCTTTGCCTACCTCT	CTTGAACCCACTTCTTCTCTGG
Ip10	GTGAGAATGAGGGCCATAGG	TTTTTGGCTAAACGCTTTCAT
Il6	GGGAAATCGTGGAAATGAGAAA	ATCCAGTTTGGTAGCATCCATC
Cxcl1	CACTCAAGAATGGTCGCGAG	GTTGTCAGAAGCCAGCGTTC
Tnf	ACTGAACTTCGGGGTGATCG	TCTTTGAGATCCATGCCGTTG
Il-1b	TGGACCTTCCAGGATGAGGACA	GTTCATCTCGGAGCCTGTAGTG
Tlr3	AGCAACAACAACATAGCCAACA	AATTCAAGATGTGGAGGTGAGA
Tlr4	ATTCAGAGCCGTTGGTGTATCT	ATTCCAGGTAGGTGTTTCTGCT
Tlr7	ATTACGGCTTCTGGACAAACTC	TATCTGTTATCACCGGCTCTCC
Tlr9	GTCCTTCAATTACCGCAAGAAG	AGATGCAGAGTGTGGAGTTTGG
Rab1a	TGGTGTGGATTTCAAGATACGAAC	GCTCCTCTGTAATAACTGGAAGTG
Rab1b	GGCAACAAGAGTGACCTCACCA	ATGCCTGCTCAACATTGGTGGC
Rnf115	GTCCCAGATGTGACTCAGGCTT	GGATTGCTACTTAGAAATGGTC
RNF115	AGCTGACAAGGAAAAGATCACA	CTAGCCACGGCACAATACAAC
RAB1A	GGGAACAAATGTGATCTGACCAC	GAAAGACTGTTCTACATTCGTTGC
